# Comparative Genomics and Metabolomics Analyses of Clavulanic Acid-Producing *Streptomyces* Species Provides Insight Into Specialized Metabolism

**DOI:** 10.3389/fmicb.2019.02550

**Published:** 2019-11-08

**Authors:** Nader F. AbuSara, Brandon M. Piercey, Marcus A. Moore, Arshad Ali Shaikh, Louis-Félix Nothias, Santosh K. Srivastava, Pablo Cruz-Morales, Pieter C. Dorrestein, Francisco Barona-Gómez, Kapil Tahlan

**Affiliations:** ^1^Department of Biology, Memorial University of Newfoundland, St. John’s, NL, Canada; ^2^Collaborative Mass Spectrometry Innovation Center, Skaggs School of Pharmacy and Pharmaceutical Sciences, University of California, San Diego, La Jolla, CA, United States; ^3^Evolution of Metabolic Diversity Laboratory, Unidad de Genómica Avanzada (Langebio), Cinvestav-IPN, Irapuato, Mexico

**Keywords:** *Streptomyces*, specialized metabolism, metabolomics, genomics, gene clusters, β-lactams, clavulanic acid

## Abstract

Clavulanic acid is a bacterial specialized metabolite, which inhibits certain serine β-lactamases, enzymes that inactivate β-lactam antibiotics to confer resistance. Due to this activity, clavulanic acid is widely used in combination with penicillin and cephalosporin (β-lactam) antibiotics to treat infections caused by β-lactamase-producing bacteria. Clavulanic acid is industrially produced by fermenting *Streptomyces clavuligerus*, as large-scale chemical synthesis is not commercially feasible. Other than *S. clavuligerus*, *Streptomyces jumonjinensis* and *Streptomyces katsurahamanus* also produce clavulanic acid along with cephamycin C, but information regarding their genome sequences is not available. In addition, the *Streptomyces* contain many biosynthetic gene clusters thought to be “cryptic,” as the specialized metabolites produced by them are not known. Therefore, we sequenced the genomes of *S. jumonjinensis* and *S. katsurahamanus*, and examined their metabolomes using untargeted mass spectrometry along with *S. clavuligerus* for comparison. We analyzed the biosynthetic gene cluster content of the three species to correlate their biosynthetic capacities, by matching them with the specialized metabolites detected in the current study. It was recently reported that *S. clavuligerus* can produce the plant-associated metabolite naringenin, and we describe more examples of such specialized metabolites in extracts from the three *Streptomyces* species. Detailed comparisons of the biosynthetic gene clusters involved in clavulanic acid (and cephamycin C) production were also performed, and based on our analyses, we propose the core set of genes responsible for producing this medicinally important metabolite.

## Introduction

**FIGURE 1:**
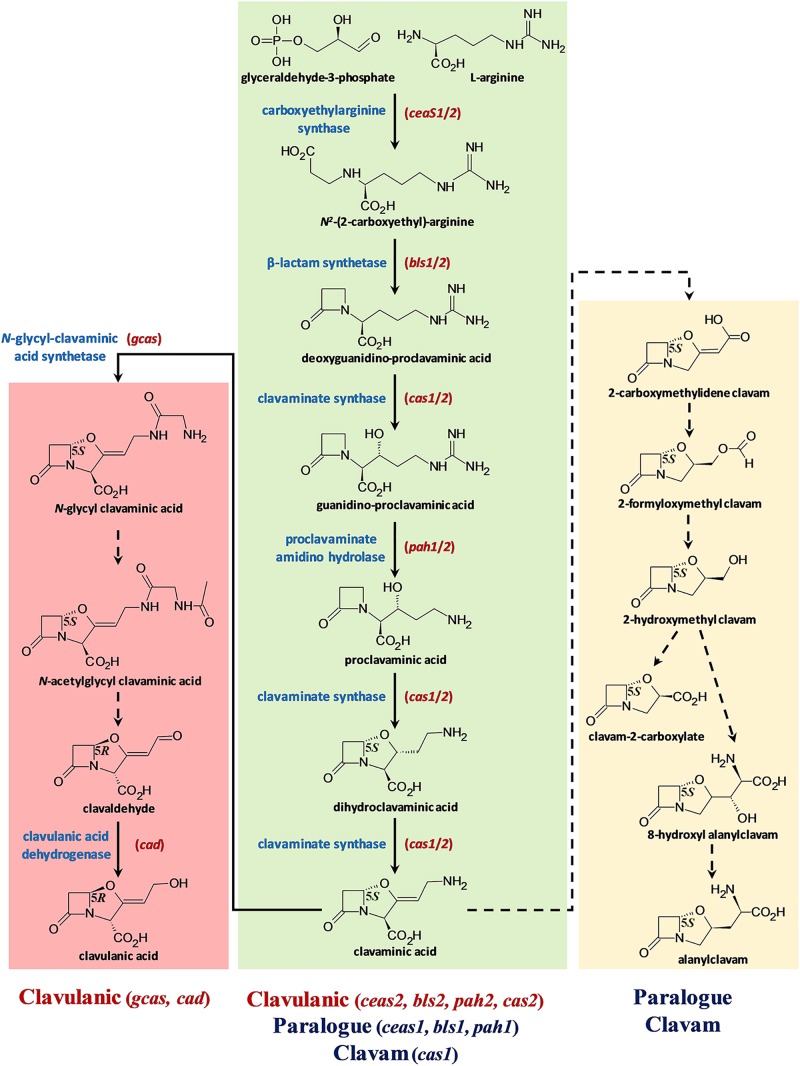
The *S. clavuligerus* clavulanic acid and 5*S* clavam biosynthetic pathways. The pathway is depicted in three parts: the central green box represents the steps shared between CA and 5*S* clavam biosynthesis, whereas the pink (left) and yellow (right) boxes indicate the late steps specific for CA or 5*S* clavam production, respectively. The solid arrows represent known reactions and broken arrows indicate uncharacterized steps, which could potentially involve more than one unknown gene product/enzyme. The names of core biosynthetic enzymes (blue) catalyzing known reactions and the respective gene(s) encoding them (red) are included where applicable. The stereochemistries (*R*/*S*) of the intermediates/products are also included along with their names. The identities of the gene clusters involved in each stage of biosynthesis is indicated at the bottom of the figure. Note that the shared part of the pathway (green) involves substitutable isozymes (CeaS, Bls, Cas, and Pah), which are encoded by two sets of genes (1 and 2) residing in three separate gene clusters. Additional genes from the respective clusters for which exact biosynthetic functions have not been assigned are not shown to simply interpretation.

Bacteria from the genus *Streptomyces* produce numerous and diverse specialized (or secondary) metabolites (SMs), many of which have medicinal applications (Baltz, 2008). Some of these SMs are also used as antibiotic adjuvants, agents administered in conjunction with antibiotics to potentiate or restore their activities against resistant bacteria (Tyers and Wright, 2019). Clavulanic acid (CA, a 5*R* clavam SM, Figure 1) is an irreversible inhibitor of certain class A and D serine β-lactamases, which are enzymes that hydrolyze β-lactam antibiotics such as the penicillins and cephalosporins to confer resistance (Drawz and Bonomo, 2010). Therefore, CA is widely used in human and veterinary medicine in combination with β-lactam antibiotics to treat otherwise resistant infections caused by β-lactamase-producing bacteria (Brown, 1986).

Clavulanic acid is industrially produced by fermenting the bacterium *Streptomyces clavuligerus* (Jensen and Paradkar, 1999; Townsend, 2002; Saudagar et al., 2008), which was first identified during screens for microorganisms capable of producing β-lactam antibiotics such as cephamycin C (Ceph-C) (Brown et al., 1976). Apart from *S. clavuligerus*, *Streptomyces jumonjinensis* and *Streptomyces katsurahamanus* are the only other species known to produce CA along with Ceph-C (Ward and Hodgson, 1993; Jensen, 2012). In addition, CA production in *S. clavuligerus* generally occurs in conjunction with Ceph-C (Romero et al., 1984; Jensen and Paradkar, 1999), even though both metabolites are products of distinct biosynthetic pathways (Hamed et al., 2013). As in the case of other Actinobacterial SMs (van der Heul et al., 2018), the regulation of CA production in *S. clavuligerus* is complex and involves cluster-situated regulators, global mechanisms, and signaling cascades (Liras et al., 2008; Song et al., 2010a; Paradkar, 2013; Ferguson et al., 2016; Álvarez-Álvarez et al., 2017). *S. clavuligerus* is also unique among the CA producers described so far due to its ability to produce the structurally related 5*S* clavams (Brown et al., 1979; Pruess and Kellett, 1983), which partially share a common biosynthetic pathway with CA (Figure 1; Egan et al., 1997; Jensen, 2012). The 5*S* clavams have the opposite stereochemistry as compared to CA and are therefore not inhibitory toward β-lactamases, but instead some of them display weak antibacterial, antifungal, or antimetabolite activities (Jensen, 2012). In comparison, some *Streptomyces* species only synthesize the 5*S* clavams but not CA, suggesting that the ability to produce clavams with both stereochemistries (5*R* and 5*S*, Figure 1) might be unique to *S. clavuligerus* (Jensen and Paradkar, 1999; Challis and Hopwood, 2003).

It is now recognized that the *Streptomyces* contain many biosynthetic gene clusters (BGCs) thought to be “cryptic or silent,” as the SMs produced by them are not known (Katz and Baltz, 2016). On average, each *Streptomyces* species contains ∼30 BGCs but only produces 3–5 SMs under laboratory conditions. Additionally, recent reports have shown that *S. clavuligerus* produced some SMs only thought to originate from plants (Álvarez-Álvarez et al., 2015), highlighting the need for thoroughly cataloging specialized metabolism, even from well-studied organisms. Due to the small number of *Streptomyces* species known to produce CA, it is of interest to determine if *S. clavuligerus*, *S. jumonjinensis*, and *S. katsurahamanus* also share other metabolic capabilities. Therefore, we sequenced the genomes of *S. jumonjinensis* and *S. katsurahamanus*, conducted comparative metabolomics analysis on the three CA producers to identify SMs, and correlated their biosynthesis with predicted BGCs wherever possible.

The described analyses also provide information on BGC content from *S. jumonjinensis* and *S. katsurahamanus*, insight that was not available previously. In *S. clavuligerus*, three separate BGCs are involved in clavam metabolite biosynthesis (Tahlan et al., 2004a). The clavulanic acid BGC is primarily associated with CA production (Jensen et al., 2000, 2004a; Li et al., 2000; Mellado et al., 2002), whereas the clavam and paralog BGCs are involved in the biosynthesis of the 5*S* clavams (Figure 1; Tahlan et al., 2007; Zelyas et al., 2008). Because of the common biosynthetic origins of CA and the 5*S* clavams, it has been suggested that there is sharing of intermediates between the pathways (Figure 1). Therefore, many gene products from the CA, clavam, and paralog BGCs contribute to the early part of the biosynthetic pathway involved in both CA and the 5S clavam production (Figure 1; Jensen, 2012; Hamed et al., 2013; Álvarez-Álvarez et al., 2018). Previous genetic mapping studies have shown that the BGCs for CA and Ceph-C are clustered together on the chromosomes of all CA producers to form “β-lactam superclusters” (Ward and Hodgson, 1993), but details about their sequences from *S. jumonjinensis* and *S. katsurahamanus* were lacking. It has been hypothesized that CA biosynthesis evolved in an ancestral 5*S* clavam producer, after it acquired the ability to produce Ceph-C by horizontal gene transfer (Challis and Hopwood, 2003). Such an arrangement leads to the coordinated biosynthesis of Ceph-C and CA, or the production of a β-lactam antibiotic and a synergistically acting β-lactamase inhibitor, respectively. The complete biosynthetic pathway leading to Ceph-C has been elucidated (Liras, 1999), but some late steps required for CA production remain unknown (Jensen, 2012; Hamed et al., 2013). Additionally, not all genes from the proposed *S. clavuligerus* CA BGC are required for CA production (Supplementary Table S1), and the exact function of many gene products remains to be deciphered (Jensen et al., 2004a; Valegård et al., 2013; Álvarez-Álvarez et al., 2018; Srivastava et al., 2019). Recently available genome sequences have revealed that CA-like BGCs (without any associated Ceph-C BGCs) are also present in other organisms such as *Streptomyces pratensis* ATCC 33331 (formerly called *Streptomyces flavogriseus*) and *Saccharomonospora viridis* DSM 43017, but neither have been shown to produce CA to date (Jensen, 2012; Álvarez-Álvarez et al., 2013). Therefore, it is still not clear as to what defines the boundaries of a functional (or minimal) CA BGC, a question that we also address in the current study.

## Materials and Methods

### Bacterial Strains, Plasmids, Media/Culture Conditions, and Molecular Methods

Bacterial strains and plasmids used in the current study are described in Table 1. All media/reagents were purchased from Fisher Scientific or VWR International (Canada). For routine analysis, *Streptomyces* cultures were maintained on International *Streptomyces* Project (ISP) medium 4 agar or were grown in Trypticase Soy Broth supplemented with 1% (w/v) soluble starch (TSB-S). Cultures for metabolite analysis were grown using glycerol, sucrose, proline, and glutamic acid (GSPG); starch asparagine (SA); soy; or TSB-S media (Romero et al., 1997; Tahlan et al., 2004b). All *Streptomyces* cultures were incubated at 28°C and liquid cultures were agitated at 250 rpm. Plasmid-bearing *Streptomyces* cultures were supplemented with appropriate antibiotics when required (Tahlan et al., 2004b), whereas *Escherichia coli* was grown and maintained as described previously (Sambrook, 2001). Standard methods were used for isolating and manipulating DNA from *E. coli* (Sambrook, 2001) and *Streptomyces* (Kieser et al., 2000; Tahlan et al., 2004b). Total RNA was isolated from *S. clavuligerus* grown on SA medium as described previously (Srivastava et al., 2019), and RT was performed using the Maxima H Minus First Strand cDNA Synthesis Kit (Thermo Scientific, United States). PCRs were carried out using the Phusion or Taq DNA polymerase kits (ThermoFisher, United States). When required, PCR products were cloned into the pGEM-T Easy vector (Promega, United States) according to the manufacturer’s instruction and were sequenced at the Centre for Applied Genomics, University of Toronto (Canada). All DNA oligonucleotide primers used in the study (Supplementary Table S2) were obtained from Integrated DNA Technologies (United States).

**TABLE 1:** Bacterial strains and plasmids used in this study.

**Strain/plasmid**	**Description^a^**	**Source/Reference**
**Bacterial strain**		
*E. coli* ESS	Indicator strain for Ceph-C bioassays	Wang et al., 2004
*E. coli* ET12567/pUZ8002	Non-methylating conjugation host carrying helper plasmid pUZ8002 (Cml^R^ and Kan^R^)	Kieser et al., 2000
*E. coli* DH5α	General laboratory cloning host	Promega
*K. pneumoniae* ATCC 15380	Indicator strain for CA bioassays (Pen^R^)	ATCC
*S. clavuligerus* ATCC27064	Wild-type CA producer	ATCC
*S. clavuligerus* Δ*nocE*	*nocE* null mutant	This study
*S. clavuligerus* pIJ8668-*ermE*p^∗^-*nocE*	*S*train constitutively expressing *nocE*	This study
*S. jumonjinensis* NRRL 5741	Wild-type CA producer	Jensen and Paradkar, 1999
*S. katsurahamanus* T272	Wild type CA producer	Jensen and Paradkar, 1999
**Plasmids**		
pGEMT-Easy	Plasmid for cloning PCR products	Promega
pIJ8668-*ermE*p^∗^	Conjugative *Streptomyces* suicide vector containing *ermE*p^∗^ for chromosomal promoter insertion (Apr^R^)	(Tahlan et al., 2017)
pIJ8668-*ermE*p^∗^*-nocE*	pIJ8668-*ermE*p^∗^ containing a portion of the 5′ end of *nocE* from *S. clavuligerus* (Apr^R^)	This study
pIJ12738	Conjugative *Streptomyces* suicide vector containing an I-SceI site for gene targeting (Apr^R^)	Fernández-Martínez and Bibb, 2014
pIJ12738-*nocE*-UP-DN	pIJ12738 containing regions upstream and downstream of *nocE* from *S. clavuligerus* (Apr^R^)	This study
pIJ12742	Plasmid expressing the Meganuclease I-SceI in *Streptomyces* for gene disruption (Apr^R^ and Tsr^R^)	Fernández-Martínez and Bibb, 2014

*^*a*^Apr^*R*^, apramycin resistance; Cml^*R*^, chloramphenicol resistance; Kan^*R*^, kanamycin resistance; Pen^*R*^, penicillin G resistance; and Tsr^*R*^, thiostrepton resistance.*

### Genome Sequencing, Gene Cluster Identification, and Bioinformatics Analyses

The *S. jumonjinensis* and *S. katsurahamanus* genomes were sequenced using Illumina MiSEQ in paired-end format with read lengths of 300 bp. A chromosomal DNA library was prepared for each organism using the PCR-based method adjusted for high GC DNA according to the manufacturer’s instructions (Illumina, United States). Raw reads were filtered with trimmomatic (Bolger et al., 2014) with a cutoff of 26 bp and a minimum length of 150 bp. The remaining reads were assembled using Velvet (Zerbino and Birney, 2008). *k*-mers from 30 to 170 were tested for selecting optimal contig length and the assembled genomes (31–46 × coverage, Supplementary Table S3) were submitted to NCBI (accession numbers: *S. jumonjinensis* NRRL 5741, VCLA00000000 and *S. katsurahamanus* T-272, VDEQ00000000). Genome completeness was calculated (Supplementary Table S3) using BUSCO (Simao et al., 2015) and QUAST (Gurevich et al., 2013). Annotations were carried out using RAST (Overbeek et al., 2014) and also manually in Artemis (Rutherford et al., 2000). Specialized metabolite (SM) biosynthetic gene clusters (BGCs) were identified using antiSMASH 4.0 (Blin et al., 2017) and polyketide synthases/nonribosomal peptide synthetase genes were predicted using PRISM 3 (Skinnider et al., 2017). The DNA sequences of *S. jumonjinensis* and *S. katsurahamanus* BGCs were manually examined for possible frame shifts and other ambiguities. In some cases, PCR amplification was performed using custom primers (Supplementary Table S2) followed by Sanger sequencing of products to verify results. The genome sequences of *S. clavuligerus* ATCC 27064 (NZ_CM000913.1, NZ_CM000914.1), *S. pratensis* ATCC 33331 (NC_016114), *S. viridis* DSM 43017 (CP001683.1), *Streptomyces* sp. M41(2017) (NZ_MWFK00000000), *Streptomyc*es sp. PAMC26508 (NC_021055), *Streptomyces* sp. NRRL S-325 (NZ_JOIW00000000), *Streptomyces* sp. NRRL B-24051 (NZ_JOAE00000000), *Streptomyces flavovirens* NRRL B-2182 (NZ_JOAB00000000), *Streptomyces fulvoviridis* NRRL ISP-5210 (NZ_JNXH00000000), and *Streptomyces olivaceus* NRRL B-3009 (NZ_JOFH00000000) were included for comparison as the latter harbor CA-like BGCs containing homologs of all genes currently known to be involved in CA production in *S. clavuligerus* (Jensen, 2012). In addition, the sequences of the Ceph-C BGCs from *Streptomyces cattleya* 8057 (NC_017586.1) and *Nocardia lactamdurans* (also known as *Amycolatopsis lactamdurans*) (Z13971.1-Z13974.1, Z21681.1-Z21686.1 and X57310.1) were also included in the analysis. Geneious 8.1.9 (Biomatters Ltd., New Zealand) was used for sequence comparisons and constructing phylogenetic trees. Protein homologs were identified using NCBI BLAST and secretory signals were predicted using the SignalP-5.0 Server (Almagro Armenteros et al., 2019).

### Preparation of the *S. clavuligerus* ΔnocE and ermEp^∗^-nocE Strains

The *S. clavuligerus nocE* gene mutant was prepared using the meganuclease I-SceI marker-less gene deletion system (Fernández-Martínez and Bibb, 2014). DNA fragments (1–1.2 kb each) containing regions immediately upstream and downstream of *nocE* from the *S. clavuligerus* chromosome were amplified using PCR along with engineered primers (Supplementary Table S2) and were separately cloned into the pGEM-T Easy vector (Table 1). The upstream fragment was released from pGEM-T Easy by digestion with *Hin*dIII and *Eco*RI and was introduced into the same sites of pIJ12738 to give pIJ12738/*nocE*-UP. The downstream fragment was then introduced into the *Eco*RI and *Xba*I sites of pIJ12738-*nocE-*UP to give pIJ12738/*nocE*-UP-DN, which functioned as the *nocE* disruption construct (Table 1). pIJ12738-*nocE*-UP-DN was conjugated into *S. clavuligerus* to obtain the apramycin-resistant single crossover strain, which was confirmed using genomic DNA PCR (Supplementary Table S2). The plasmid pIJ12742 expressing the I-SceI meganuclease (Table 1) was then conjugated into *S. clavuligerus* pIJ12738-*nocE*-UP-DN to obtain apramycin and thiostrepton resistant exconjugants, which were made to undergo sporulation at 28°C without any selection to facilitate double homologous recombination and loss of pIJ12738 from the chromosome. Spore stocks were prepared and re-streaked onto ISP-4 plates without selection and incubated for 5 days at 37°C to promote the loss of temperature-sensitive pIJ12742. This led to the isolation of the apramycin and thiostrepton-sensitive *S. clavuligerus* Δ*nocE* mutant, which was verified using genomic DNA PCR (Supplementary Table S2).

To prepare an *S. clavuligerus* strain constitutively expressing *nocE* (Table 1), the *ermE*p^∗^ promoter (Bibb et al., 1985) was inserted upstream of the gene in the *S. clavuligerus* chromosome. A 1.1-kb DNA fragment from the 5′ end of the gene was amplified by PCR (Supplementary Table S2) and was cloned into pGEM-T Easy. The insert was re-isolated as an *Nde*I and *Eco*RI fragment and was ligated with similarly digested pIJ8668-*ermE*p^∗^ to give pIJ8668-*ermE*p^∗^*-nocE* (Table 1), which was introduced into wt *S. clavuligerus* by conjugation. This resulted in the *S. clavuligerus ermE*p^∗^*-nocE* strain, which was confirmed using genomic DNA PCR (Supplementary Table S2) and was used to examine the effect of constitutively expressing *nocE* in *S. clavuligerus*.

### Bioassays and Bacterial Growth Measurement

The production of CA and Ceph-C in culture supernatants was detected (and quantified in the case of Ceph-C) using bioassays employing indicator organisms (Table 1), as described previously (Paradkar and Jensen, 1995; Wang et al., 2004). Growth in liquid cultures was determined using a modified diphenylamine colorimetric method to measure DNA content (Zhao et al., 2013) and statistical analysis (ANOVA repeated measure) was performed using R 3.4.3. To assess for growth characteristics on solid media, 10-fold dilutions of a spore stock (4 × 10^4^ spores/μl) were prepared, and 5 μl of which were spotted onto two different agar media (SA and TSB-S with 1.5% agar). The plates were then incubated at 28°C and visually scored for growth over a 7-day period.

### Liquid Chromatography–Mass Spectrometry (LC-MS and LC-MS/MS) Analysis

The production of clavam metabolites in 96-h broth cultures was analyzed by targeted LC-MS after imidazole derivatization using an XTerra column (2.1 × 150 mm, 3.5 μm, 125 Å; Waters Scientific, United States) as described previously (Srivastava et al., 2019). Untargeted metabolomics was conducted using bacteria grown on solid media. One hundred microliters of a standardized spore stock (4 × 10^4^ spores/μl) of each species was used to inoculate agar plates in duplicate, and each plate was extracted using 15 ml of methanol or ethyl acetate. Two milliliters of each extract was dried, resuspended in 130 μl of 70% methanol containing 0.2 μM of amitriptyline (internal standard), and transferred to a 96-well plate, which was centrifuged at 2000 rpm for 15 min at 4°C. One hundred microliters of each sample was then transferred to a new 96-well plate for LC-MS/MS analysis. Samples were analyzed using a Vanquish UHPLC System coupled Q Exactive Hybrid Quadrupole-Orbitrap Mass Spectrometer (Thermo Scientific, United States). Chromatographic separation was performed in mixed mode (allowing weak anion/cation exchange) on a Scherzo SM-C18 column (2 × 250 mm, 3 μm, 130 Å; Imtakt, United States) maintained at 40°C. Ten microliters of each sample was injected for analysis and the mobile phase consisted of (A) 0.1% formic acid in water and (B) 0.1% formic acid in acetonitrile. Chromatography was performed at a flow rate of 0.5 ml/min using the following program: 0–5 min, 98% A; 5–8 min, gradient of 98–50% A (or 50% B); 8–13 min, gradient 50–100% B; 13–14.00 min, 100% B; 14–14.10 min, 100–2% B; 14.10–18 min, 2% B.

Mass spectrometry was performed using a heated electrospray ionization source (heater temperature, 370°C and capillary temperature, 350°C) in either positive or negative ionization mode (± 3000.0 V; S-lens RF, 55; sheath gas flow rate, 55; and auxiliary gas flow rate, 20). MS^1^ and MS^2^ scans (at 200 *m*/*z*) were acquired from 0.48 to 16.0 min at a resolution of 35,000 and 17,500, respectively, for the 100–1500 *m*/*z* range. The automatic gain control (AGC) target value and maximum injection time were set at 5 × 10^5^ and 150 ms. Up to four MS^2^ scans in data-dependent mode were acquired for most abundant ions per duty cycle, with a starting value of 70 *m*/z, and exclusion parameter of 10 s. Higher-energy collision-induced dissociation was performed with a normalized collision energy of 20, 35, and 50 eV. The apex trigger mode was used (2–7 s) and the isotopes were excluded. Inclusion lists of ions for molecules observed in *Streptomyces* extracts were generated from the Dictionary of Natural Products^1^ and the StreptomeDB (Lucas et al., 2013), and were used for prioritizing the acquisition of their MS^2^ when observed. The raw LC-MS/MS data files were converted to .mzXML format using ProteoWizard (Adusumilli and Mallick, 2017). All metabolomics MS data have been deposited on the MassIVE public repository^2^ under the accession number MSV000083835.

### MS Data Annotation and Analysis

Molecular networks were generated using positive and negative ionization mode data in GNPS (Wang et al., 2016). The resulting networks were visualized in Cytoscape (Shannon et al., 2003), allowing nodes associated with uninoculated media controls to be removed. Annotations were first obtained by matching spectra in public libraries (Wang et al., 2016), including NIST17^3^. Library annotations were manually validated using mirror plots (maximum ion mass accuracy = 5 ppm) corresponding to level 2 annotation based on the Minimum Standard Initiative (Spicer et al., 2017). The data were deposited to the GNPS library (CCMSLIB00005435954-CCMSLIB00000531493), which enabled the annotation of putative tunicamycin derivatives (CCMSLIB00005435941-42) and lyngbyatoxin (CCMSLIB00005435954-55) using molecular networks. In some cases, Sirius 4.0.1 was used to confirm the molecular formulas of certain predicted metabolites (Böcker et al., 2009).

To generate a heat map using the *S. clavuligerus* wt, Δ*nocE*, and *ermEp^∗^-nocE* strains, feature-based detection and alignment of positive mode ionization data were performed (parameters: MS^1^ noise level of 25000, MS^2^ noise level of 1000) using the MZmine 2 toolbox (v2.39) (Pluskal et al., 2010). Chromatograms were built using the ADAP module (parameters: min group size in # of scans = 4, group intensity threshold = 700,000, min highest intensity = 100,000, max *m/z* tolerance = 10 ppm), which were then deconvoluted (parameters: S/N threshold = 10.0, min feature height = 7000000, coefficient/area threshold = 60.0, peak duration range = 0.01–0.5 min, RT wavelet range = 0.01–0.1 s). Fragmentation spectra were paired with deconvoluted peaks using 0.02 Da and 0.2 min windows, and LC-MS features were annotated using the Peak-Grouping module (parameters: deisotope = true, remove features without isotope pattern = false, minimal intensity for interval selection = 0.1, minimal intensity overlap = 0.7, minimal correlation = 0.7). Features were aligned in the JoinAligner module (parameters: ppm tolerance = 7, weight for *m/z* = 75.0, retention time tolerance = 0.5 min, weight for RT = 25.0; require same charge state = false, require same ID = false, compare isotope pattern = false). The aligned peaklist was filtered with the row filter module to keep only features with at least two isotopic ions, two occurrences, and at least one MS^2^ spectrum before gap filling (parameters: intensity = 5%, ppm window = 5, retention time tolerance = 0.15). The aligned peaklist containing 3149 features was exported as a .CSV file, and the spectral data as .MGF files using the GNPSExport module for further processing. The signal intensities of the features (.CSV) were normalized to that of an internal standard (*m/z* 278.189; retention time, 9.2 min) and only 1684 features with an intensity 3-fold higher than in experimental controls (uncultivated media) were retained. MetaboAnalyst4.0 (Chong et al., 2018) was used to perform the hierarchal clustering, which was visualized as a heat map.

## Results and Discussion

**FIGURE 2:**
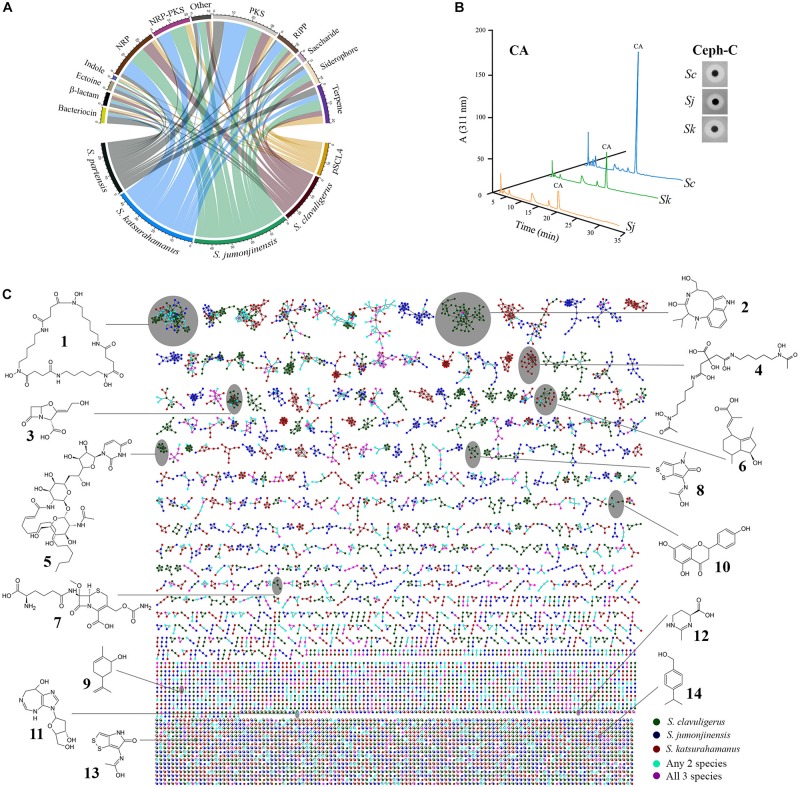
Biosynthetic gene cluster (BGC) content and metabolomics analysis of clavulanic acid (CA)-producing *Streptomyces* species (*S. clavuligerus*, *S. jumonjinensis*, and *S. katsurahamanus*). **(A)** Circular chord diagram representing all predicted BGC classes present in the respective *Streptomyces* species. *S. pratensis* was included for comparison as the bacterium contains a CA-like BGC, but does not produce the metabolite. The sequence of the giant linear plasmid pSCL4 from *S. clavuligerus* was also included separately due to the presence of multiple BGCs on it. The lower arc represents genomes/plasmid, while the upper arc represents different classes of BGCs and the color-coded ribbons connecting them indicate the presence of a BGC in the specific species. **(B)** Detection of CA and cephamycin C (Ceph-C) in 96-h SA culture supernatants of *S. clavuligerus* (*Sc*), *S. jumonjinensis* (*Sj*), and *S. katsurahamanus* (*Sk*) using LC-MS (after imidazole derivatization) and bioassays (inset), respectively. The peak corresponding to CA in HPLC chromatograms is noted and the zones of inhibition in the inset panel demonstrate relative amounts of Ceph-C production. **(C)** Metabolic network constructed using *S. clavuligerus*, *S. jumonjinensis*, and *S. katsurahamanus* culture extracts (culture conditions and details are described in the section “Materials and Methods”). The network is color-coded according to source organism (bottom right legend), where each node depicts a mass spectrum and edges represent the relationship between different nodes. Structures of natural products detected in the extracts at high confidence in the three species are shown and the clade in the network containing the node corresponding to the respective metabolite is also indicated. 1, desferrioxamine E; 2, (−)-indolactam V; 3, clavulanic acid; 4, arthrobactin; 5, tunicamycin C2; 6, hydroxyvalerenic acid; 7, cephamycin C; 8, thiolutin; 9, (−)-carveol; 10, naringenin; 11, pentostatin, 12, ectoine; 13, holomycin; and 14, cuminyl alcohol.

**FIGURE 3:**
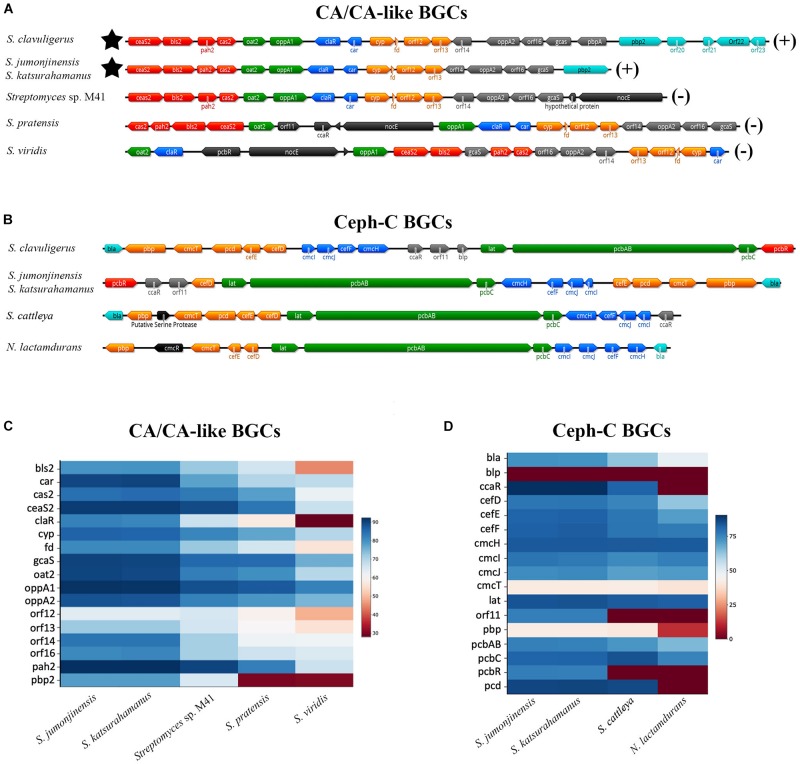
Organization of CA/CA-like **(A)** and Ceph-C **(B)** BGCs in select CA-producing/non-producing *Streptomyces* species. The CA non-producers *Saccharomonospora viridis* **(A,C)** and *Nocardia* (or *Amycolatopsis*) *lactamdurans* **(B,D)** were also included for comparison as both are phylogenetically distinct from the *Streptomyces*. **(A,B)** The architecture of respective BGCs from the described organisms showing their gene content and relative organization. Genes are color-coded based on known or predicted transcriptional units. **(A)** The star symbol represents the location of the Ceph-C BGC (if present) and the CA production status (±) of each organism is indicated on the right. **(B)** All species included are Ceph-C and CA producers except for *S. cattleya* and *N. lactamdurans*, which only produce the former. **(C,D)** Relative identities of protein products from the CA/CA-like **(C)** and Ceph-C **(D)** BGCs of described organisms as compared to corresponding homologs from *S. clavuligerus*. The legend on the right shows colors indicating percent identities between respective gene products.

Three *Streptomyces* species are known to produce CA, but details about the involved BGCs are only available for the genome sequenced industrial producer, *S. clavuligerus* (Medema et al., 2010; Song et al., 2010b; Cao et al., 2016). Therefore, we sequenced the genomes of the other two CA producers, *S. jumonjinensis* and *S. katsurahamanus* (Table 1), for comparative studies. The published genome sequence of *S. pratensis* ATCC 33331 was also included during some of the analyses (Figure 2A), as it contains a CA-like BGC (Figures 3A,C), and has been shown not to produce the metabolite under tested conditions (Álvarez-Álvarez et al., 2013). Examination of the *S. jumonjinensis* and *S. katsurahamanus* genomes revealed that they each contain 49 and 44 known or predicted SM BGCs (Table 2 and Supplementary Table S4), respectively, which is much higher than the average number found in many *Streptomyces* species. Additionally, *S. clavuligerus* contains 43 SM BGCs, although re-sequencing of its genome suggests that it may contain many more (Hwang et al., 2019). This prompted us to further investigate the specialized metabolic capabilities of the three CA producers to determine similarities or differences between these microorganisms.

**TABLE 2:** Genome features relevant to specialized metabolism in *S. jumonjinensis* and *S. katsurahamanus* as compared to *S. clavuligerus* (CA producer) and *S. pratensis* (CA non-producer).

**Feature**	***S. jumonjinensis* NRRL 5741**	***S. katsurahamanus* T272**	***S. clavuligerus* ATCC27064**	***S. pratensis* ATCC 33331**
Genome size (Mbp)	8.47^a^	7.25^a^	8.56	7.34
Coding sequences	7423	6123	7281	6537
SM BGCs^b^ (PKS/NRPS)^c^	49 (8/18)	44 (9/9)	43 (10/9)	27 (5/9)

*^*a*^ Estimated sizes based on sequence analysis from the current study. ^*b*^ Specialized metabolite (SM) biosynthetic gene clusters (BGCs) were predicted using antiSMASH 4.0. ^*c*^ Polyketide synthase (PKS) and nonribosomal peptide synthetase (NRPS) genes were predicted using PRISM 3.*

### SM-BGCs and Metabolism in *S. clavuligerus*, *S. jumonjinensis*, and *S. katsurahamanus*

Detailed analysis of the *S. jumonjinensis* and *S. katsurahamanus* genome sequences using antiSMASH 4.0 (Blin et al., 2017) and manual curation showed that both organisms contain numerous BGCs for diverse SMs (Figure 2A and Supplementary Table S4). Therefore, *S. clavuligerus*, *S. jumonjinensis*, and *S. katsurahamanus* were grown on SA, GSPG, and TSB-S media for assessing CA/Ceph-C production (Figure 2B and Supplementary Figure S1) and for preparing methanol/ethyl acetate extracts for liquid chromatography–tandem mass spectrometry (LC-MS/MS)-based metabolomics. The MS/MS data obtained from both positive and negative ionization mode were used to build a molecular network (Figure 2C), and metabolites were annotated by matching spectra against public libraries corresponding to level 2 annotation based on the Metabolomics Standard Initiative (Wang et al., 2016). During the analysis, ions corresponding to CA ([M-H]^–^, *m*/*z* 198.039), Ceph-C ([M-H]^–^, *m*/*z* 445.104) and numerous other SMs were also detected in extracts from one or more *Streptomyces* species (Figure 2C, Supplementary Tables S5, S6), some of which are discussed below.

The desferrioxamines (Figure 2C) comprise a group of nonpeptide hydroxamate siderophores produced by many bacteria (Barona-Gómez et al., 2004), including *S. clavuligerus* (Álvarez-Álvarez et al., 2017). In the current study, ions corresponding to desferrioxamine E (Nocardamine, [M + H]^+^, *m*/*z* 601.356) and desferrioxamine B (Desferal, [M + H]^+^, *m*/*z* 561.361) were detected in extracts from *S. clavuligerus*/*S. jumonjinensis* and *S. clavuligerus*/*S. katsurahamanus*, respectively (Supplementary Table S5). Desferrioxamine E exhibits antitumor activity (Kalinovskaya et al., 2011), while desferrioxamine B is used in therapy for secondary iron overload disease (Olivieri and Brittenham, 1997). We also identified BGCs in *S. clavuligerus*, *S. jumonjinensis*, and *S. katsurahamanus* (Supplementary Table S4) that have high degrees of similarity (80–100%) with BGCs from known desferrioxamine producers such as *Streptomyces griseus* (Yamanaka et al., 2005; Ohnishi et al., 2008) and *Streptomyces coelicolor* A3(2) (Bentley et al., 2002; Barona-Gómez et al., 2004). The siderophore arthrobactin (Figure 2C) was also detected in *S. katsurahamanus* extracts ([M + H]^+^, *m*/*z* 477.256) (Supplementary Table S5), but since the genes responsible for arthrobactin production are not known (Burrell et al., 2012), we were unable to identify an associated BGC in this organism. However, our analysis showed that *S. clavuligerus*, *S. jumonjinensis*, and *S. katsurahamanus* each contain additional siderophore-like BGCs of unknown function (Supplementary Table S4), which could potentially be involved in the production of such metabolites. Ectoine is another commonly produced metabolite that helps bacteria survive extreme osmotic stress (Sadeghi et al., 2014), and it was detected ([M + H]^+^, *m*/*z* 143.082) in extracts from all three CA-producing species (Figure 2C and Supplementary Table S5). In addition, *S. clavuligerus*, *S. jumonjinensis*, and *S. katsurahamanus* contain BGCs that are similar to the known ectoine BGC from *Streptomyces anulatus* (previously called *Streptomyces chrysomallus*) (Prabhu et al., 2004). Since the desferrioxamines and ectoine are produced by many Actinomycetes and are involved in general cellular growth/survival processes (Challis, 2005; Czech et al., 2018), finding them in culture extracts from the three CA producers in the current study was not surprising.

*Streptomyces clavuligerus* is a known producer of the dithiolopyrrolone antibiotic holomycin (Kenig and Reading, 1979) and the associated BGC has been identified in this organism (Li and Walsh, 2010). In the current study, holomycin ([M + H]^+^, *m*/*z* 214.994) and thiolutin (another dithiolopyrrolone, [M + H]^+^, *m*/*z* 229.010) were detected in extracts from *S. clavuligerus*, but not in those from *S. jumonjinensis* or *S. katsurahamanus* (Figure 2C and Supplementary Table S5). Recently, a dithiolopyrrolone with the same molecular weight as thiolutin (predicted to be *N*-propionylholothin) was also detected in extracts from *S. clavuligerus* strains lacking the giant linear plasmid pSCL4 (Álvarez-Álvarez et al., 2017). Since holomycin and thiolutin (Figure 2C), and the respective BGCs involved in their biosynthesis (from *S. clavuligerus* and *Saccharothrix algeriensis* NRRL B-24137, respectively), are very similar (Supplementary Table S4), it is possible that a single pathway in *S. clavuligerus* produces both metabolites. It has also been reported that there is some sort of cross regulation between CA and holomycin production in *S. clavuligerus* (de la Fuente et al., 2002; Álvarez-Álvarez et al., 2017). Our results showed that *S. jumonjinensis* and *S. katsurahamanus* lack dithiolopyrrolone BGCs (Supplementary Table S4) and therefore do not have a similar link between holomycin and CA production as observed in *S. clavuligerus*.

We also detected certain nucleoside SMs during the current analysis (Figure 2C). For example, the purine nucleoside pentostatin, which is also used as an anticancer agent (Dillman, 2004), was identified ([M + 2H]^2+^, *m*/*z* 135.066) in *S. clavuligerus* extracts (Figure 2C, Supplementary Table S5). A putative pentostatin-like BGC was recently shown to be present in *S. clavuligerus* (Wu et al., 2017), but production of the metabolite has not been reported in this organism previously. Therefore, our results suggest that the *S. clavuligerus* pentostatin BGC can be activated under laboratory conditions. The tunicamycins also comprise a mixture of related nucleoside antibiotics, some of which (A, B, C, and I) were detected in extracts from *S. clavuligerus* (Figure 2C and Supplementary Table S5), but not in those from *S. jumonjinensis* or *S. katsurahamanus*. *S. clavuligerus* is a known producer of tunicamycin and the BGC involved in its production has been identified (Kenig and Reading, 1979; Chen et al., 2010). In addition, certain derivatives of tunicamycin I with different acyl chains were detected in *S. clavuligerus* extracts recently (Martínez-Burgo et al., 2019), which were also present in our samples (Supplementary Table S5). Our results demonstrated that *S. jumonjinensis* and *S. katsurahamanus* do not possess tunicamycin BGCs (Supplementary Table S4), further distinguishing *S. clavuligerus* from the other CA producers due to its ability to produce such nucleoside SMs.

Metabolomics analysis also revealed the presence of certain plant-associated SMs in the *Streptomyces* extracts. It was recently shown that *S. clavuligerus* produces the citrus flavonoid naringenin and the genes involved in the production of this metabolite were also identified (Álvarez-Álvarez et al., 2015). Naringenin exhibits antibacterial, antifungal, and anticancer activities (Rauha et al., 2000; Kanno et al., 2005), and its production by a bacterium was unexpected since it was previously isolated from plants only (Álvarez-Álvarez et al., 2015). We detected naringenin (Figure 2C, [M-H]^–^, *m*/*z* 271.062) in extracts from *S. clavuligerus* and *S. jumonjinensis*, but not from *S. katsurahamanus* (Supplementary Table S5). In addition, the genes involved in naringenin production were also found in both *S. jumonjinensis* and *S. katsurahamanus* (Supplementary Table S4), suggesting that the metabolite might be produced at undetectable levels in *S. katsurahamanus* or that the genes are not expressed in this species under the conditions tested. Also detected in all three *Streptomyces* extracts were the plant-associated monoterpenes, carveol ([M-H_2_O + H]^+^, *m*/*z* 135.117), and cuminyl alcohol ([M-H_2_O + H]^+^, *m*/*z* 133.101), whereas hydroxyvalerenic acid (another plant terpene, [2M-H]^–^, *m*/*z* 499.307) was found in *S. clavuligerus* extracts only (Figure 2C and Supplementary Table S5). The pathways involved in the production of the latter three metabolites are not fully known (Wong et al., 2018), however, *S. clavuligerus*, *S. jumonjinensis*, and *S. katsurahamanus* possess many terpene-like BGCs of unknown function, which could potentially be involved in their biosynthesis (Supplementary Table S4). Therefore, our results suggest that certain *Streptomyces* also harbor the capacity to produce carveol, cuminyl alcohol, and hydroxyvalerenic acid along with naringenin, a finding that can be potentially exploited for further development.

The indole alkaloid, (−)-indolactam V is a protein kinase C activator (Heikkila and Akerman, 1989) and functions as an intermediate during the biosynthesis of other SMs in certain Actinomycetes (Abe, 2018). We detected (−)-indolactam V (Figure 2C, [M-CO + H]^+^, *m*/*z* 274.191) and some of its alkylated derivatives in extracts from *S. clavuligerus*, but not in those from *S. jumonjinensis* or *S. katsurahamanus* (Supplementary Table S5). The genes normally associated with (−)-indolactam V biosynthesis could not be identified in the current study, warranting further investigation into its production in *S. clavuligerus*. Other metabolites were also detected during the analysis (Supplementary Table S6), but we were unable to find details about their biosynthesis in bacteria or predict associated BGCs, and therefore we did not include them in the discussion. In addition, *S. jumonjinensis* and *S. katsurahamanus* contain several BGCs related to known pathways for which products could not be detected (Supplementary Table S4). For example, there is an NRPS-containing BGC in *S. jumonjinensis* that is 100% similar to the BGC in *Streptomyces* sp. DSM 11171, which produces the antiviral metabolite feglymycin (Supplementary Table S4; Gonsior et al., 2015). We also identified indole-associated BGCs in *S. clavuligerus* and *S. jumonjinensis* (Supplementary Table S4), which are similar to the one from *Streptomyces* sp. TP-A0274 responsible for producing the anticancer agent staurosporine (Onaka et al., 2002). Similarly, BGCs for polycyclic tetramate macrolactams (PTMs, NRP/PKs) are present in both *S. jumonjinensis* and *S. katsurahamanus*, which are 100% similar to a SGR-PTM BGC from the known producer *S. griseus* (Supplementary Table S4; Luo et al., 2013). PTMs possess antifungal and antioxidant properties, and cryptic PTM-like BGCs are commonly found in *Streptomyces* genomes (Zhang et al., 2016). Moreover, BGCs for many other classes of SMs including enediynes (Rudolf et al., 2016) and the ribosomally synthesized and post-translationally modified peptides (RiPPs) (Hetrick and van der Donk, 2017) were also identified in *S. jumonjinensis* and *S. katsurahamanus* (Supplementary Table S4), but further work is required to detect their production in these organisms. In the current study, >14,000 molecular nodes were obtained using MS-based metabolomics and GNPS analysis (Figure 2C), but only 10% could be annotated by matching spectra with available libraries. Therefore, many of the unannotated nodes could represent products of so-called “cryptic” BGCs, a situation that should change over time as databases are populated with more spectra from authentic samples.

### Comparative Sequence Analysis of CA-BGCs From *Streptomyces* Species

In addition to analyzing the overall SM production capabilities of CA producers, we were also interested in specifically examining the BGCs involved in β-lactam biosynthesis from *S. jumonjinensis* and *S. katsurahamanus* for comparison with *S. clavuligerus* (Figure 3). The genome sequences of *S. jumonjinensis* and *S. katsurahamanus* revealed that they both contain identical CA and Ceph-C BGCs (Figure 3), but lack the clavam and paralog gene clusters (Supplementary Table S4). This would explain why they do not produce the 5*S* clavams as compared to *S. clavuligerus* (Jensen, 2012). The results further confirm that intact 5*S* clavam and paralog BGCs are not essential for CA production (Figure 1), since both *S. jumonjinensis* and *S. katsurahamanus* can produce the metabolite (Figure 2B and Supplementary Figure S1). The paralog gene cluster from *S. clavuligerus* contains second copies of certain genes (*ceaS1, bls1*, and *pah1*) from the CA BGC (Jensen et al., 2004b; Tahlan et al., 2004b), which encode enzymes involved in the early shared stages of CA and 5*S* clavam biosynthesis (Figure 1). It has also been shown that the remaining un-duplicated genes from the paralog gene cluster and almost all genes from the clavam gene cluster (except one; *cas1*) are exclusively involved in 5*S* clavam production (Mosher et al., 1999; Tahlan et al., 2007; Zelyas et al., 2008). Therefore, our results provide additional support for the hypothesis that the clavam and paralog gene clusters are associated with 5*S* clavam biosynthesis, and that some gene products from the two clusters augment CA production in *S. clavuligerus* by contributing to a common pool of precursors (Figure 1; Jensen, 2012; Hamed et al., 2013). Although, it should be noted that in *S. clavuligerus*, there is some cross regulation between the chromosomal CA and plasmid-borne paralog gene clusters (Kwong et al., 2013; Álvarez-Álvarez et al., 2017), which is again not expected to occur in the other two CA producers since they only contain the CA BGC. This also highlights the complexity of the regulatory pathways controlling CA and 5*S* clavam production in *S. clavuligerus* (Liras et al., 2008). For this reason, we focused our analysis and discussion on the comparison of biosynthetic genes (and BGCs), instead of regulation. In the current study, CA production levels in *S. jumonjinensis* and *S. katsurahamanus* could never match those observed in wt *S. clavuligerus*, whereas all three species produced Ceph-C at comparable levels (Figure 2B). It has been previously suggested that higher CA yields in *S. clavuligerus* might be explained in part by increased precursor supply for biosynthesis due to the presence of the paralog and clavam gene clusters in this species (Figure 1). In addition, enhanced levels of biosynthetic gene expression could be another reason why *S. clavuligerus* is currently the preferred industrial producer and was first identified in screens for β-lactamase inhibitors, as higher CA yields would make it easier to detect during assays (Jensen, 2012).

Closer examination of the CA BGCs from *S. jumonjinensis* and *S. katsurahamanus* showed that they each contain most of the genes from the corresponding *S. clavuligerus* BGC in the same order, except that *orf18* (*pbpA*), *orf20*, *orf21*, *orf22*, and *orf23* are absent (Figure 3A). *pbpA* is predicted to encode a high-molecular-weight penicillin-binding protein (PBP), but its role in CA production remains unknown (Jensen et al., 2004a). Previous studies have also shown that disruption of *orf19* (*pbp2*) (Jensen et al., 2004a), *orf20* (cytochrome P-450) (Song et al., 2009), *orf21* (putative sigma factor), *orf22* (sensor kinase), or *orf23* (response regulator) (Fu et al., 2019a) in *S. clavuligerus* does not abolish CA or Ceph-C production (Song et al., 2009; Supplementary Table S1). Since the respective genes are not present in *S. jumonjinensis* and *S. katsurahamanus* (Figure 3A), it is apparent that they are not part of the core BGC required for biosynthesis, but instead have accessory roles in *S. clavuligerus*. In a previous study, it was also shown that the expression of *orf18–21* was not significantly affected in a *S. clavuligerus* mutant defective in ClaR, the cluster-situated regulator responsible for controlling CA biosynthesis (Martínez-Burgo et al., 2015). Therefore, we propose that the core CA BGC comprises *ceaS2* (encoding carboxyethylarginine synthase), *gcas* (encoding *N*-glycyl-clavaminic acid synthetase), and the intervening genes (Figure 3A, and Supplementary Table S1).

The CA and Ceph-C BGCs in *S. jumonjinensis* and *S. katsurahamanus* also form “β-lactam superclusters” as observed in *S. clavuligerus*, which agrees with previous restriction mapping studies (Ward and Hodgson, 1993). The linkage of the Ceph-C and CA BGCs in *S. clavuligerus*, *S. jumonjinensis*, and *S. katsurahamanus*, and the coordinated production of the two metabolites in *S. clavuligerus* (Pérez-Llarena et al., 1997), provides further evidence for the simultaneous acquisition of the two BGCs by producing species. It has been proposed that the CA BGC might have evolved by the duplication of an ancestral 5*S* clavam BGC and the acquisition of the ability to produce Ceph-C in the same organism (Challis and Hopwood, 2003). Such a situation led to the selection for the ability to produce a β-lactamase inhibitor, resulting in the assembly of the currently known CA BGC, and the formation of the β-lactam supercluster (Challis and Hopwood, 2003). Our results showed that the Ceph-C BGCs from *S. jumonjinensis* and *S. katsurahamanus* are identical to each other, but differ slightly from those present in *S. clavuligerus* and other Ceph-C-producing Actinobacteria (Figures 3B,D). The positions of genes forming individual operons (or transcriptional units) in all three CA producers is very similar (except for the location of *cefD*), but the relative arrangement of operons is different in *S. jumonjinensis* and *S. katsurahamanus* as compared to *S. clavuligerus* (Figure 3B). In addition, the Ceph-C BGCs of *S. jumonjinensis*, *S. katsurahamanus* and other previously reported Ceph-C producers (other than *S. clavuligerus*) (Liras et al., 1998) do not contain *blp* (Figure 3B), which encodes a product resembling β-lactamase inhibitory proteins (Blip), but has been shown to lack any such activity (Gretes et al., 2009). Previous studies have shown that disruption of *blp* does not affect Ceph-C or CA production in *S. clavuligerus* (Alexander and Jensen, 1998; Thai et al., 2001). Therefore, *blp* does not seem to a part of the core Ceph-C BGC since *S. jumonjinensis*, *S. katsurahamanus*, and other species shown in Figure 3B can still produce the metabolite in its absence. Another noticeable feature of Ceph-C BGCs from the three CA producers is the presence of *pcbR*, which is missing from the homologous BGCs of species that only produce Ceph-C, but not CA (Figure 3B). PcbR resembles PBPs (Paradkar et al., 1996), but it is not essential for Ceph-C biosynthesis since it is not present in the BGCs of all organisms capable of producing the metabolite (Figure 3B, more details below).

Overall, the “β-lactam superclusters” from *S. clavuligerus*, *S. jumonjinensis*, and *S. katsurahamanus* are very similar to each other (Figures 3C,D). In comparison, CA-like BGCs from non-producers are markedly different, and do not form “β-lactam superclusters” as they lack Ceph-C BGCs (Jensen, 2012). The non-producers (including some *Streptomyces*) are also phylogenetically distinct from CA-producing species (Supplementary Figure S2), and their CA-like BGCs show three distinct patterns in terms of gene content and arrangement (Figure 3A). Many organisms in the database contain CA-like BGCs identical to the one found in *S. pratensis*, whereas we could only find one example each of the types present in *Streptomyces* sp. M41 and *S. viridis*, respectively (Figure 3A). In addition, CA-like BGCs from *S. pratensis* and *S. viridis* contain the *pcbR*, *orf11*, and *nocE* genes (Álvarez-Álvarez et al., 2013), which are not present in the CA BGCs of *S. clavuligerus*, *S. jumonjinensis*, or *S. katsurahamanus* (Figure 3A). Interestingly, *pcbR* and *orf11* are included in the Ceph-C BGCs of CA producers, whereas *nocE* is located elsewhere on the chromosome in the three *Streptomyces* species (Figure 3B). As mentioned earlier, *pcbR* encodes a PBP involved in β-lactam resistance (Paradkar et al., 1996), whereas *orf11* encodes a predicted protein of unknown function. Previous reports have shown that disruption of neither *pcbR* nor *orf11* in *S. clavuligerus* affected Ceph-C or CA production (Paradkar et al., 1996; Alexander and Jensen, 1998), suggesting that they are not required for the biosynthesis of the respective metabolites.

The presence of *nocE* homologs in CA producers and in the CA-like BGCs of all non-producers is intriguing (Figure 3A), as they are similar to a gene from the nocardicin A monobactam BGC of *Nocardia uniformis* (Gunsior et al., 2004). The *nocE* genes are predicted to encode proteins containing C-terminal SGNH/GDSL hydrolase family domains, which are normally associated with esterases or lipases (Upton and Buckley, 1995), but their function during β-lactam metabolite biosynthesis is not obvious. The disruption of *nocE* in *N. uniformis* does not affect nocardicin A production (Davidsen and Townsend, 2009), but the role of the gene in β-lactam-producing *Streptomyces* has not been examined to date.

### Examination of the Function of nocE in *S. clavuligerus*

**FIGURE 4:**
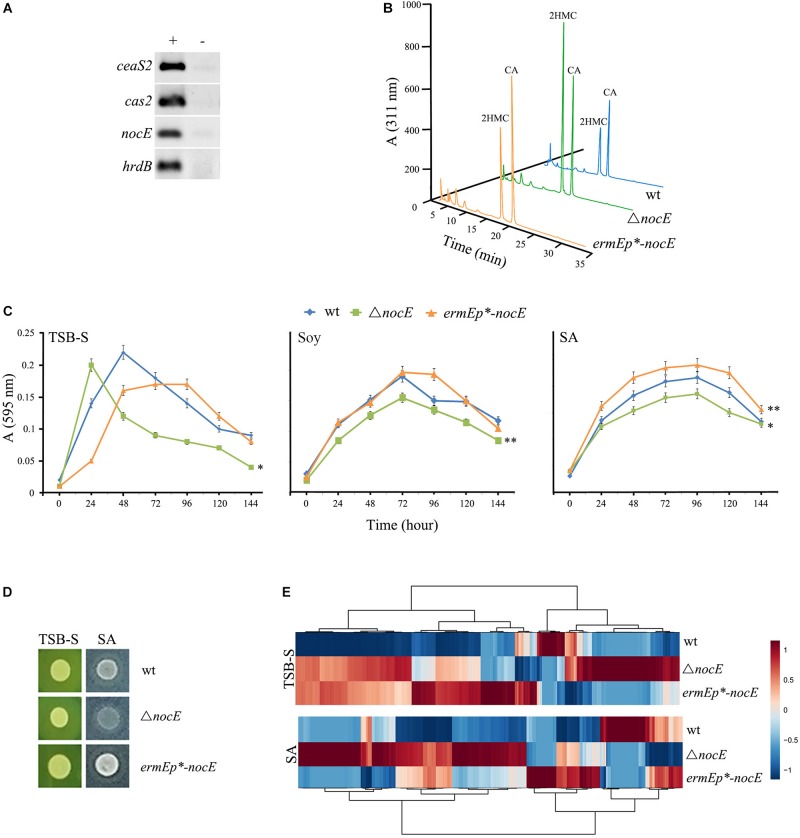
Examination of the function of *nocE* in *S. clavuligerus*. **(A)** RT-PCR analysis (+) of RNA isolated from 96-h *S. clavuligerus* SA cultures showing the expression of *nocE* during CA production. Transcription of *ceaS2* and *cas2* was used as a reporter for CA-BGC expression, whereas that of the constitutively expressed *hrdB* was used as a control. Negative controls (−) consisted of RNA samples subjected to PCR without undergoing RT. **(B)** LC-MS analysis of imidazole derivatized 96-h soy culture (different media from Figure 2B) supernatants form the *S. clavuligerus* wt, Δ*nocE*, and *ermEp*^∗^-*nocE* (constitutive expression) strains to assess CA and 5*S* clavam metabolite production. **(C,D)** Growth characteristics of the *S. clavuligerus* wt, Δ*nocE*, and *ermE*p^∗^*-nocE* strains in broth **(D)** or agar **(D)** cultures under different nutritional conditions, where (^∗^) and (^∗∗^) indicate *p* values of less than 0.05 and 0.001, respectively. **(E)** Comparative metabolomics of the *S. clavuligerus* wt, Δ*nocE* and *ermE*p^∗^*-nocE* strains grown on two different media as shown in panel **(D)**. The heat map was constructed by hierarchical clustering of ∼1000 statically significant features to show overall differences between the three strains.

In previous studies, every gene from the proposed CA BGC of *S. clavuligerus* (Figure 3A) was systematically disrupted (Supplementary Table S1), to determine if it had any effect on CA or Ceph-C production. It has been suggested that *nocE* might have some role during CA biosynthesis in *S. clavuligerus*, but since the gene is not part of the CA BGC, a mutant has not been prepared and analyzed to date (Jensen, 2012). Therefore, the function of *nocE* was examined in the model CA producer, *S. clavuligerus*. RT-PCR analysis of RNA isolated from wt *S. clavuligerus* grown in SA medium demonstrated that *nocE* is temporally expressed along with *ceaS2* and *cas2* (Figure 4A), genes that are essential for CA biosynthesis (Figure 1). However, when *S. clavuligerus* strains were prepared in which *nocE* was either deleted (Δ*nocE*) or constitutively expressed (*ermE*p^∗^-*nocE*) (Table 1), the production of CA, 5*S* clavams, or Ceph-C was found to be unaffected (Figure 4B and Supplementary Figure S3), demonstrating that the gene is not required for β-lactam metabolite production in *S. clavuligerus*. The predicted lipase/esterase-like domain present in NocE is also found in hydrolytic enzymes from other *Streptomyces* species, some of which are known to be secreted (Wei et al., 1995; Vujaklija et al., 2002). Closer examination of the predicted NocE amino acid sequence from *S. clavuligerus* suggested that it is also a secreted protein, as it contains a highly conserved N-terminal Sec-signal sequence (*p* > 0.9) (Almagro Armenteros et al., 2019). These findings further ruled out the direct involvement of NocE in CA production, which occurs in the cytoplasm, and suggested that NocE might have some other exocellular hydrolytic function instead. Therefore, the *S. clavuligerus* wt, Δ*nocE*, and *ermE*p^∗^-*nocE* strains were assessed for growth under different nutritional condition using TSB-S (rich), soy (complex fermentation), or SA (defined fermentation) media (Figure 4C). It was observed that the growth of the *S. clavuligerus*Δ*nocE* mutant was significantly reduced in each medium tested, whereas that of the *ermE*p*^∗^-nocE* strain was enhanced in SA medium only, when compared to the wt strain (Figure 4C). The growth of the three strains was also assessed on TSB-S and SA agar, which again showed that the *S. clavuligerus* Δ*nocE* mutant did not grow as well as the other strains in the latter medium (Figure 4D). To examine the influence of *nocE* on primary metabolism in *S. clavuligerus*, the wt, Δ*nocE*, and *ermE*p^∗^-*nocE* strains were grown on TSB-S and SA agar for metabolomics analysis, which showed marked differences in overall metabolite levels between the respective strains (Figure 4E). Furthermore, metabolomics analysis showed that SM production in *S. clavuligerus* was unaffected in the Δ*nocE* mutant as compared to the wt strain. Therefore, based on all evidence collected so far, it seems plausible that NocE could have some extracellular role in nutrient acquisition in *S. clavuligerus*, but like *pcbR* and *orf11*, it is not required for CA or Ceph-C production under the tested conditions.

## Conclusion

To summarize, we have shown that *S. clavuligerus*, *S. jumonjinensis*, and *S. katsurahamanus* contain numerous BGCs and that they synthesize many SMs, including the plant-associated metabolites, naringenin, and valerenic acid. It is possible that genes encoding enzymes for the synthesis of plant-associated metabolites are present in *Streptomyces* genomes, but they are not easily identified due to their organization, since some of them do not form BGCs (Álvarez-Álvarez et al., 2015; Nybo et al., 2017). In addition, plants normally produce metabolites like valerenic acid in low amounts, and for this reason, their heterologous production has been recently attempted in *Saccharomyces* and *Escherichia coli* (Nybo et al., 2017; Wong et al., 2018). The finding that certain *Streptomyces* species can synthesize these metabolites naturally could provide future avenues for their overproduction in a native host. Our results also show similarities and differences in the overall specialized metabolic capabilities of CA-producing *Streptomyces* species under different nutritional conditions, which, to the best of our knowledge, is the first report on the subject. Although the current study did not examine or address regulation, we would like to point out that many of the genes known to control Ceph-C and CA production in *S. clavuligerus* are also conserved in the two other producers (Liras et al., 2008; Ferguson et al., 2016; Fu et al., 2019b). It has been noted that deciphering the complete CA biosynthetic pathway in *S. clavuligerus* is challenging due to the presence of the 5*S* clavam biosynthetic pathway. The current report provides a framework for future studies on CA biosynthesis using *S. jumonjinensis* or *S. katsurahamanus* as models due to the absence of such competing or overlapping pathways in these organisms. Our analyses have also allowed us to propose the core group of genes involved in CA biosynthesis and have helped us to rule out the involvement of *nocE* and other genes in the production of this important metabolite.

## Data Availability Statement

The datasets generated and/or analyzed during this study can be found in the NCBI sequence database (ncbi.nlm.nih.gov/genome) and the MassIVE public repository (massive.ucsd.edu). All accession numbers are provided in the Materials and Methods section.

## Author Contributions

KT contributed conception, resources, and supervision. FB-G and PD provided reagents, resources, and supervision for genomics and metabolomics analysis, respectively. MM and PC-M performed the genome sequencing and annotation. NA and BP conducted the described comparative genomics analysis. NA prepared and analyzed the *S. clavuligerus nocE* mutant and overexpression strains. NA and SS prepared extracts for LC-MS/MS analysis, which was performed by L-FN. AS and L-FN carried out the metabolomics analysis and compound annotation. NA and MM wrote the first draft of the manuscript, whereas BP, AS, and L-FN wrote specific sections. NA, BP, AS, L-FN, FB-G, and KT contributed to manuscript revision.

## Conflict of Interest

The authors declare that the research was conducted in the absence of any commercial or financial relationships that could be construed as a potential conflict of interest.

## Footnotes

1 http://dnp.chemnetbase.com

2 massive.ucsd.edu

3 www.nist.gov
