# Supplementary material for: Comparative Genomics and Metabolomics Analyses of Clavulanic Acid-Producing Streptomyces Species Provides Insight Into Specialized Metabolism
Source: Front Microbiol. 2019 Nov 8;10:2550. doi: 10.3389/fmicb.2019.02550 (PMC6856088; doi:10.3389/fmicb.2019.02550)

Supplementary Table S1. Clavulanic acid (CA) and cephamycin C (Ceph-C) production in *S. clavuligerus* mutants with defects in genes from the clavulanic acid biosynthetic gene cluster. Phenotypes for some *S. clavuligerus* gene mutants that have homologues in clavulanic acid-like gene clusters of non-producers are also included.

Gene	Product (function)	Metabolite production [a]		Reference[b]
		Ceph-C	CA	
ceaS1/2[c]	Carboxyethylarginine synthase (biosynthesis)	Yes	No	(Pérez-Redondo et al., 1999) (Jensen et al., 2000) (Tahlan et al., 2004)
bls1/2[c]	β-Lactam synthetase (biosynthesis)	Yes	No	(Bachmann et al., 1998) (Jensen et al., 2000) (Tahlan et al., 2004)
pah1/[c]	Proclavaminic acid amidinohydrolase (biosynthesis)	Yes	No	(Aidoo et al., 1994) (Jensen et al., 2004b)
cas1/2[c]	Clavaminic acid synthase (biosynthesis)	Yes	No	(Mosher et al., 1999) (Jensen et al., 2000)
oat1/2[c]	Ornithine acetyltransferase	Yes	Yes	(de la Fuente et al., 2004) (Tahlan et al., 2004)
oppA1	Oligopeptide transporter	Yes	No	(Lorenzana et al., 2004) (Jensen et al., 2000) (Álvarez-Álvarez et al., 2018)
claR	Transcriptional activator (regulation)	Yes	No	(Paradkar et al., 1998) (Pérez-Redondo et al., 1998) (Jensen et al., 2000) (Martínez-Burgo et al., 2015)
car (cad)	Clavaldehyde reductase or dehydrogenase (biosynthesis)	Yes	No	(Jensen et al., 2000)
cyp (orf10)	Cytochrome P-450 (biosynthesis)	Yes	No	(Jensen et al., 2000) (Li et al., 2000) (Mellado et al., 2002)
fd (orf11)	Ferredoxin	Yes	70-80% of wt	(Jensen et al., 2004a)
cpe (orf12)	β-Lactamase-like protein (biosynthesis)	Yes	No	(Jensen et al., 2004a) (Li et al., 2000) (Valegård et al., 2013) (Srivastava et al., 2019)
orf13	Membrane transport protein	Yes	No	(Mellado et al., 2002) (Jensen et al., 2004a)
cbg (orf14)	Acetyltransferase (biosynthesis)	Yes	No	(Mellado et al., 2002) (Jensen et al., 2004a)
oppA2 (orf15)	Oligopeptide transporter (biosynthesis)	Yes	No	(Mellado et al., 2002) (Jensen et al., 2004a) (Lorenzana et al., 2004) (Álvarez-Álvarez et al., 2018)
orf16	N-Acetyltranferase (biosynthesis)	Yes	No	(Mellado et al., 2002) (Jensen et al., 2004a)
gcas (orf17)	N-glycyl-clavaminic acid synthetase (biosynthesis)	Yes	No	(Mellado et al., 2002) (Jensen et al., 2004a) (Arulanantham et al., 2006)
pbpA (orf18)	Penicillin binding protein	NA[d]	NA[d]	(Mellado et al., 2002) (Jensen et al., 2004a)

pbp2 (orf19)	Penicillin binding protein	Yes	Yes	(Mellado et al., 2002)<br>(Jensen et al., 2004a)
orf20	Cytochrome P-450	Yes	Yes	(Jensen, 2012)<br>(Shrestha et al., 2017)
orf21	RNA polymerase σ factor (regulation)	Yes	Yes	(Song et al., 2009)<br>(Jnawali et al., 2011)
orf22	Two-component system histidine kinase (regulation)	Yes	Yes	(Song et al., 2009)<br>(Fu et al., 2019)
orf23	Two-component system response regulator (regulation)	47% of wt	40% of wt	(Jnawali et al., 2008)<br>(Song et al., 2009)<br>(Fu et al., 2019)
ccaR	Transcriptional activator (regulation)	No	No	(Alexander and Jensen, 1998)
pcbR	Penicillin binding protein (resistance)	Yes	Yes	(Paradkar et al., 1996)
orf11	Unknown	Yes	Yes	(Alexander and Jensen, 1998)
nocE	Lipases/esterases	Yes	Yes	**This study**

[a] >95% level of production when compared to wild type *S. clavuligerus* is reported as "Yes" and <5% production is reported as "No"

[b] References:

Aidoo, K.A., Wong, A., Alexander, D.C., Rittammer, R.A., and Jensen, S.E. (1994). Cloning, sequencing and disruption of a gene from *Streptomyces clavuligerus* involved in clavulanic acid biosynthesis. *Gene* 147(1), 41-46. doi: 10.1016/0378-1119(94)90036-1.

Alexander, D.C., and Jensen, S.E. (1998). Investigation of the *Streptomyces clavuligerus* cephamycin C gene cluster and its regulation by the CcaR protein. *J Bacteriol* 180(16)**,** 4068-4079.

Álvarez-Álvarez, R., Rodríguez-García, A., Martínez-Burgo, Y., Martín, J.F., and Liras, P. (2018). Transcriptional Studies on a *Streptomyces clavuligerus* oppA2 Deletion Mutant: *N*-Acetylglycyl-Clavaminic Acid Is an Intermediate of Clavulanic Acid Biosynthesis. *Appl Environ Microbiol* 84(22). doi: 10.1128/AEM.01701-18.

Arulanantham, H., Kershaw, N.J., Hewitson, K.S., Hughes, C.E., Thirkettle, J.E., and Schofield, C.J. (2006). ORF17 from the clavulanic acid biosynthesis gene cluster catalyzes the ATP-dependent formation of N-glycyl-clavaminic acid. *J Biol Chem* 281(1), 279-287. doi: 10.1074/jbc.M507711200.

Bachmann, B.O., Li, R., and Townsend, C.A. (1998). beta-Lactam synthetase: a new biosynthetic enzyme. *Proc Natl Acad Sci U S A* 95(16), 9082-9086. doi: 10.1073/pnas.95.16.9082.

de la Fuente, A., Martín, J.F., Rodríguez-García, A., and Liras, P. (2004). Two proteins with ornithine acetyltransferase activity show different functions in *Streptomyces clavuligerus*: Oat2 modulates clavulanic acid biosynthesis in response to arginine. *J Bacteriol* 186(19), 6501-6507. doi: 10.1128/JB.186.19.6501-6507.2004.

Fu, J., Qin, R., Zong, G., Liu, C., Kang, N., Zhong, C., et al. (2019). The CagRS Two-Component System Regulates Clavulanic Acid Metabolism via Multiple Pathways in *Streptomyces clavuligerus* F613-1. *Front Microbiol* 10, 244. doi: 10.3389/fmicb.2019.00244.

Jensen, S.E. (2012). Biosynthesis of clavam metabolites. *J Ind Microbiol Biotechnol* 39(10), 1407-1419. doi: 10.1007/s10295-012-1191-0.

Jensen, S.E., Elder, K.J., Aidoo, K.A., and Paradkar, A.S. (2000). Enzymes catalyzing the early steps of clavulanic acid biosynthesis are encoded by two sets of paralogous genes in *Streptomyces clavuligerus*. *Antimicrob Agents Chemother* 44(3), 720-726. doi: 10.1128/aac.44.3.720-726.2000.

Jensen, S.E., Paradkar, A.S., Mosher, R.H., Anders, C., Beatty, P.H., Brumlik, M.J., et al. (2004a). Five additional genes are involved in clavulanic acid biosynthesis in *Streptomyces clavuligerus*. *Antimicrob Agents Chemother* 48(1)**,** 192-202. doi: 10.1128/aac.48.1.192-202.2004.

Jensen, S.E., Wong, A., Griffin, A., and Barton, B. (2004b). *Streptomyces clavuligerus* has a second copy of the proclavaminate amidinohydrolase gene. *Antimicrob Agents Chemother* 48(2)**,** 514-520. doi: 10.1128/aac.48.2.514-520.2004.

Jnawali, H.N., Liou, K., and Sohng, J.K. (2011). Role of sigma-factor (*orf21*) in clavulanic acid production in *Streptomyces clavuligerus* NRRL3585. *Microbiol Res* 166(5)**,** 369-379. doi: 10.1016/j.micres.2010.07.005.

Jnawali, H.N., Oh, T.J., Liou, K., Park, B.C., and Sohng, J.K. (2008). A two-component regulatory system involved in clavulanic acid production*. J Antibiot (Tokyo)* 61(11), 651-659. doi: 10.1038/ja.2008.92.

Li, R., Khaleeli, N., and Townsend, C.A. (2000). Expansion of the clavulanic acid gene cluster: identification and *in vivo* functional analysis of three new genes required for biosynthesis of clavulanic acid by *Streptomyces clavuligerus*. *J Bacteriol* 182(14)**,** 4087-4095. doi: 10.1128/jb.182.14.4087-4095.2000.

Lorenzana, L.M., Pérez-Redondo, R., Santamarta, I., Martín, J.F., and Liras, P. (2004). Two oligopeptide-permease-encoding genes in the clavulanic acid cluster of *Streptomyces clavuligerus* are essential for production of the beta-lactamase inhibitor. *J Bacteriol* 186(11), 3431-3438. doi: 10.1128/JB.186.11.3431-3438.2004.

Martínez-Burgo, Y., Álvarez-Álvarez, R., Rodríguez-García, A., and Liras, P. (2015). The Pathway-Specific Regulator ClaR of *Streptomyces clavuligerus* Has a Global Effect on the Expression of Genes for Secondary Metabolism and Differentiation. *Appl Environ Microbiol* 81(19)**,** 6637-6648. doi: 10.1128/AEM.00916-15.

Mellado, E., Lorenzana, L.M., Rodríguez-Sáiz, M., Díez, B., Liras, P., and Barredo, J.L. (2002). The clavulanic acid biosynthetic cluster of *Streptomyces clavuligerus*: genetic organization of the region upstream of the car gene. *Microbiology* 148(Pt 5), 1427-1438. doi: 10.1099/00221287-148-5-1427.

Mosher, R.H., Paradkar, A.S., Anders, C., Barton, B., and Jensen, S.E. (1999). Genes specific for the biosynthesis of clavam metabolites antipodal to clavulanic acid are clustered with the gene for clavaminate synthase 1 in *Streptomyces clavuligerus*. *Antimicrob Agents Chemother* 43(5)**,** 1215-1224.

Paradkar, A.S., Aidoo, K.A., and Jensen, S.E. (1998). A pathway-specific transcriptional activator regulates late steps of clavulanic acid biosynthesis in *Streptomyces clavuligerus*. *Mol Microbiol* 27(4), 831-843. doi: 10.1046/j.1365-2958.1998.00731.x.

Paradkar, A.S., Aidoo, K.A., Wong, A., and Jensen, S.E. (1996). Molecular analysis of a beta-lactam resistance gene encoded within the cephamycin gene cluster of *Streptomyces clavuligerus*. *J Bacteriol* 178(21)**,** 6266-6274. doi: 10.1128/jb.178.21.6266-6274.1996.

Pérez-Redondo, R., Rodríguez-García, A., Martín, J.F., and Liras, P. (1998). The claR gene of *Streptomyces clavuligerus*, encoding a LysR-type regulatory protein controlling clavulanic acid biosynthesis, is linked to the clavulanate-9-aldehyde reductase (car) gene. *Gene* 211(2):311-21. doi: 10.1016/s0378-1119(98)00106-1.

Pérez-Redondo, R., Rodríguez-García, A., Martín, J.F., and Liras, P. (1999). Deletion of the pyc gene blocks clavulanic acid biosynthesis except in glycerol-containing medium: evidence for two different genes in formation of the C3 unit. *J Bacteriol* 181(22), 6922-6928.

Song, J.Y., Kim, E.S., Kim, D.W., Jensen, S.E., and Lee, K.J. (2009). A gene located downstream of the clavulanic acid gene cluster in *Streptomyces clavuligerus* ATCC 27064 encodes a putative response regulator that affects clavulanic acid production. *J Ind Microbiol Biotechnol* 36(2)**,** 301-311. doi: 10.1007/s10295-008-0499-2.

Shrestha, B., Darsandhari, S., Kim, T.S., and Sohng, J.K. (2017). Investigating the Role of CYP 450 (orf20) Involved in Clavulanic acid Biosynthetic Pathway in *Streptomyces clavuligerus* NRRL 3585. *18th International Symposium on the Biology of Actinomycetes*, At ICC JEJU, KOREA.

Srivastava, S.K., King, K.S., AbuSara, N.F., Malayny, C.J., Piercey, B.M., Wilson, J.A., et al. (2019). *In vivo* functional analysis of a class A beta-lactamase-related protein essential for clavulanic acid biosynthesis in *Streptomyces clavuligerus*. *PLoS One* 14(4), e0215960. doi: 10.1371/journal.pone.0215960.

Tahlan, K., Park, H.U., Wong, A., Beatty, P.H., and Jensen, S.E. (2004). Two sets of paralogous genes encode the enzymes involved in the early stages of clavulanic acid and clavam metabolite biosynthesis in *Streptomyces clavuligerus*. *Antimicrob Agents Chemother* 48(3)**,** 930-939. doi: 10.1128/aac.48.3.930-939.2004.

Valegård, K., Iqbal, A., Kershaw, N.J., Ivison, D., Généreux, C., Dubus, A., et al. (2013). Structural and mechanistic studies of the orf12 gene product from the clavulanic acid biosynthesis pathway. *Acta Crystallogr D Biol Crystallogr* 69(Pt 8), 1567-1579. doi: 10.1107/S0907444913011013.

[c] There are two copies each of these genes in the clavulanic acid, clavam and/or paralogue gene clusters of *S. clavuligerus*, and phenotypes of double disruption mutants are reported

[d] NA: not applicable. Mutants could not be obtained and the gene was porposed to be essential for survival in *S. clavuligerus*

Supplementary Table S2. Sequences of oligonucleotide primers used in the current study and their details.

Name	Sequence (5' – 3')	Product size	Description
nocE-KO-UP-F2<br>nocE-KO-UP-R2	AAGCTTCCCTGGCTGAAACCCTATGG<br>GAATTCGCGCTTGGATCTGCTCAAAG	1224 bp	Primers for amplification of the upstream region of *nocE* from *S. clavuligerus* to prepare pIJ12738-*nocE*-UP-DN
nocE-KO-DN-F<br>nocE-KO-DN-R2	GAATTCCTGCCGTCGATGAAGTCCTT<br>TCTAGACACCAAGGCGATCCTCTACC	1221 bp	Primers for amplification of the downstream region of *nocE* from *S. clavuligerus* to prepare pIJ12738-*nocE*-UP-DN
nocE-KO-UP-F2<br>nocE-KO-DN-R2	AAGCTTCCCTGGCTGAAACCCTATGG<br>TCTAGACACCAAGGCGATCCTCTACC	2445 bp	Primers for confirming upstream and downstream regions of *nocE* in *S. clavuligerus* pIJ12738-*nocE*-UP-DN
Sc-nocE-F2<br>Sc-nocE-R1	GTCGAGAAGCTCCCGTACCA<br>CGGTAGCCGTGGACCATCTT	1787 bp	Primers for detection of *nocE* in *S. clavuligerus* pIJ12738-*nocE*-UP-DN
nocE-UPDN-ID-F<br>nocE-UPDN-ID-R	GTCTGAACCACTTTCGCAGC<br>GTGAAGTGGCATGGCGAATC	439 bp	Primers for confirming the presence of upstream and downstream regions of *nocE* in *S. clavuligerus* Δ*nocE*
Sc-nocE-F1<br>nocE-ID-R	GCCGACGAGAAGGACGGTTA<br>CAGCTTGTTGGTGAAGGTGC	156 bp	Primers for confirming deletion of *nocE* in *S. clavuligerus* Δ*nocE*
nocE-KN-F<br>nocE-KN-R	CATATGGAATTTCCCCGGACTCC<br>GAATTCACCTCACCACCGGTCAGATA	1088 bp	Primers for amplification of the 5' end of *nocE* from *S. clavuligerus* to prepare pIJ8668-*ermE*p*-*nocE*
ermEp-F<br>nocE-K-R	GATATCGGTACCAGCCCGAC<br>GCGCTTGGATCTGCTCAAAG	578 bp	Primers for confirming the insertion of *ermE*p* in *S. clavuligerus ermE*p*-*nocE*
Sc-nocE-F1<br>nocE-ID-R	GCCGACGAGAAGGACGGTTA<br>CAGCTTGTTGGTGAAGGTGC	156 bp	Primers for RT-PCR of *nocE* from *S. clavuligerus*
cas2-O73<br>cas2-O74	GCAAGCGGCTGGTGATGG<br>GGTCTCCGAGGACAGGTAGTGC	143 bp	Primers for RT-PCR of *cas2* from *S. clavuligerus*
ceaS2-F<br>ceaS2-R	ATCGACTTCGTTCTGACCCG<br>GGTGTCGTTCGGGAAGATGT	213 bp	Primers for RT-PCR of *ceaS2* from *S. clavuligerus*
hrdB-4F<br>hrdB-4R	CGCGGCATGCTCTTCCT<br>AGGTGGCGTACGTGGAGAAC	109 bp	Primers for RT-PCR of *hrdB* from *S. clavuligerus*
Sj-pcbC-cmcH-F<br>Sj-pcbC-cmcH-R	AACTGCGGTACGTACATGGG<br>CCACATCGACTGGAACGTGT	1089 bp	Primers for PCR amplification and sequencing of the *pcbC-cmcH* regions from the Ceph-C BGCs of *S. jumonjinensis* and *S. katsurahamanus*

Sj-or11-lat-F<br>Sj-or11-lat-R	ACCACGACGACATGGTCAC<br>GTACCTGAACTGGCGGGAAT	1904 bp	Primers for PCR amplification and sequencing of the *orf11-lat* regions from the Ceph-C BGCs of *S. jumonjinensis* and *S. katsurahamanus*
Sj-pcbR-ccaR-F<br>Sj-pcbR-ccaR-R	CTGGATGATCGGCTACCAGG<br>GAAGCGAGAAATCGCCGTTG	685 bp	Primers for PCR amplification and sequencing of the *pcbR-ccaR* regions from the Ceph-C BGCs of *S. jumonjinensis* and *S. katsurahamanus*
Sj-lat-pcbAB-F<br>Sj-lat-pcbAB-R	GAGGCAACCTCGCCGATATG<br>TCCCTGAGCGTGGTGTAGT	705 bp	Primers for PCR amplification and sequencing of the *lat-pcbAB* regions from the Ceph-C BGCs of *S. jumonjinensis* and *S. katsurahamanus*
Sk-pcb74-bla-F<br>Sk-pcb74-bla-R	CAAGAAGGGCCAGGTTCTCG<br>CAATCTGCTGATGCGCGAC	917 bp	Primers for PCR amplification and sequencing of the *pcb74-bla* regions from the Ceph-C BGCs of *S. jumonjinensis* and *S. katsurahamanus*
Sj-cmcI-cefE-F<br>Sj-cmcI-cefE-R	GTGGTAAGCCGGGTCTTCTC<br>GCTCCTCTCATAACCGGTGG	996 bp	Primers for PCR amplification and sequencing of the *cmcI-cefE* region from the Ceph-C BGC of *S. jumonjinensis*
Sk-cmcI-cefE-F<br>SK-cmcI-cefE-R	GCCGATCTGGACAGTACGTT<br>ATACGGCCGAAATACTGCGT	1070 bp	Primers for PCR amplification and sequencing of the *cmcI-cefE* region from the Ceph-C BGC of *S. katsurahamanus*
pcbAB-Sj-F<br>pcbAB-Sj-R	CTGGAACAGCAGCGGCAA<br>AGGTGTCCTCCAGCATGAAC	1773 bp	Primers for PCR amplification and sequencing of *pcbAB* from the Ceph-C BGC of *S. jumonjinensis*
pcbAB-Sk-F<br>pcbAB-Sk-R	CCGTCAACACGATGAACAGC<br>AGTTGGAGAACGACAGCAGG	1023 bp	Primers for PCR amplification and sequencing of *pcbAB* from the Ceph-C BGC of *S. katsurahamanus*

Supplementary Table S3. Details of genome sequence assemblies for *S. jumonjinensis* and *S. katsurahamanus* from the current study.

Property/Attribute	*S. jumonjinensis*	*S. katsurahamanus*
Assembly length (bp)	8465075	7243866
Coverage after assembly (fold)[a]	46	31
GC content (ratio)	0.71	0.71
Total number of contigs	209	411
N50	91474	37935
L50	28	51
Largest contig	385731	204440
Genome completeness (BUSCO %)[b]	98	98

[a] Indicates actual fold coverage based on estimated genome size.

[b] Calculated using the Benchmarking Universal Single-Copy Orthologs (BUSCO) software (Simao et al., 2015).

References:

Simao, F.A., Waterhouse, R.M., Ioannidis, P., Kriventseva, E.V., and Zdobnov, E.M. (2015). BUSCO: assessing genome assembly and annotation completeness with single-copy orthologs. *Bioinformatics* 31(19), 3210-3212. doi: 10.1093/bioinformatics/btv351.

Supplementary Table S4. Known/predicted specialized metabolite (SM) biosynthetic gene clusters (BGCs) in *S. clavuligerus* (*Sc*), *S. jumonjinensis* (*Sj*), *S. katsurahamanus* (*Sk*) and *S. pratensis* (*Sp*) as determined using antiSMASH.

SM type	BGC[a]	Function of product	BGC in respective species (% similarity)[a,b]				MIBiG BGC-ID[d]
			Sc[c]	*Sj*	*Sk*	*Sp*	
**β-lactam**	Alanylclavam	Antifungal	100 (SMCp13)	NP[e]	NP	NP	BGC0000841
	Carbapenem MM4550	Antibiotic	NP	NP	NP	65	BGC0000842
	5*S* Clavams	Antifungal	81 (SMC9)	NP	NP	NP	BGC0000843
	**Clavulanic acid**	β-lactamase inhibitor	**75 (SMC10)**	70	**70**	20	BGC0000845
**Butyrolactone**	Butyrolactone-like BGC (*Sj*, cluster 9)	Unknown	NP	100	NP	NP	NA[f]
	Butyrolactone-like BGC (Sc, pSCL4[g], cluster 8)		100 (SCLAV_p0810 - 0816)	NP	NP	NP	NA
	Butyrolactone-like BGC (*Sp*, cluster 27)		NP	NP	NP	100	NA
**Ectoine**	**Ectoine**	Osmolyte	**100 (SCLAV_1073 – 1083)**	**100**	**100**	100	BGC0000853
**Indole**	Staurosporine	Antifungal/antitumor	NP	93	NP	NP	BGC0000825
			94 (SMCp14)	NP	NP	NP	BGC0000826
**Melanin**	Melanin	Protective pigment	100 (SCLAV_3894 – 3903)	100	100	100	BGC0000911
**NRPS[h]**	A-503083	Antibiotic	NP	3	NP	NP	BGC0000288
	Chloroeremomycin	Antibiotic	NP	NP	33	NP	BGC0000322
	Coelichelin	Siderophore (peptide)	NP	NP	NP	90	BGC0000325
	Daptomycin	Antibiotic (lipopeptide)	12 (SMC5, SMCp10 and SMCp11)	9	9	NP	BGC0000336
	Feglymycin	Antibiotic/antiviral	NP	100	47	NP	BGC0001233
	**Holomycin**	Antibiotic/antitumor	**100 (SMC18)**	NP	NP	NP	BGC0000373

NRPS[h]	Indigoidine	Antioxidant/antimicrobial	40 (SMCp24)	NP	NP	NP	BGC0000375
	Leinamycin	Antimicrobial/antitumor	NP	2	NP	NP	BGC0001101
	Maduropeptin	Antitumor	NP	15	NP	NP	BGC0001008
	Marformycins	Anti-infective	NP	NP	NP	12	BGC0001214
	Nanchangmycin	Antibiotic	30 (SMC13)	NP	NP	NP	BGC0000105
	Nucleocidin	Antibiotic/anti-trypanosome	47 (SMC19)	NP	NP	NP	BGC0001387
	Pristinamycin	Antibiotic	NP	NP	17	NP	BGC0000952
	Ristocetin	Antibiotic	NP	10	NP	NP	BGC0000418
	Skyllamycin	Antitumor	NP	14	NP	NP	BGC0000429
	SW-163	Antitumor	NP	NP	7	NP	BGC0000434
	UK-68,597	Antibiotic	NP	17	NP	NP	BGC0001178
	NRPs-like BGC (*Sk*, cluster23)	Unknown	NP	NP	100	NP	NA
	NRPs-like BGC (*Sk*, cluster 30)		NP	NP	100	NP	NA
	NRPs-like BGC (*Sc*, cluster 6)		100 (SMC7)	NP	NP	NP	NA
NRPS/PKS[i]	Cyclindrospermopsin	Cyanotoxin	NP	66	14	NP	BGC0000979
	Didemnin	Antiviral/immunosuppressant	NP	18	NP	NP	BGC0000985
	Lidamycin	Antibiotic/antitumor	NP	34	34	NP	BGC0001397
	Guadinomine	Antibiotic	7 (SMCp6)	NP	NP	NP	BGC0000998
	Kosinostatin	Antibiotic/antitumor	NP	13	NP	NP	BGC0001073
	Lankacidin	Antibiotic/antitumor	NP	20	NP	NP	BGC0001100
	Rapamycin	Immunosuppressant	7 (SMCp22 and SMCp23)	NP	NP	NP	BGC0001040
	Sporolide	Antiviral	34 (SMCp17 to SMCp21)	NP	NP	NP	BGC0000150
	Zorbamycin	Antibiotic/antitumor	NP	4	4	6	BGC0001058
NRPS/T1PKS[j]	Eponemycin	Angiogenesis inhibitor /antibiotic	NP	NP	14	NP	BGC0000345
	Rifamycin	Antibiotic (anti-mycobacterial)	NP	5	NP	NP	BGC0000137
	SGR PTMs	Antimicrobial/antioxidant	NP	100	100	NP	BGC0001043

Nucleoside	A201A	Antibiotic	15 (SMC14)	NP	NP	NP	BGC0000873
	A-503083	Antibiotic	NP	3	NP	NP	BGC0000288
	**Tunicamycin**	Antibiotic	**92 (SCLAV_4276 – 4295)**	NP	NP	NP	BGC0000880
**Other**	(*Sj*, Cluster 4)	Unknown	NP	100	NP	NP	NA
**PKS**	Cinerubin B	Unknown	NP	NP	NP	31	BGC0000212
	Granaticin	Antibiotic	NP	NP	8	NP	BGC0000227
	PM100117/PM100118	Antitumor	NP	NP	8	NP	BGC0001359_c 2
	RK-682	Tyrosine phosphatase inhibitor	NP	54	54	NP	BGC0000140
	Steffimycin	Antitumor	NP	NP	NP	19	BGC0000273
	Tautomycin	Phosphatase inhibitor	NP	6	NP	NP	BGC0000159
	Tetronasin	Antibiotic	NP	NP	NP	11	BGC0000163
	Vicenistatin	Antibiotic	NP	NP	NP	60	BGC000167
	Viguiepinol	Antispasmodic	NP	NP	26	NP	BGC0000286
**RiPP[k] (Bacteriocin)**	Bacteriocin-like BGC (*Sj*, cluster 25; *Sk*, cluster25)	Unknown	NP	100	80	NP	NA
	Bacteriocin-like BGC (*Sc*, cluster 17)		100 (SCLAV_4854 – 4865)	NP	NP	NP	NA
	Bacteriocin-like BGC (*Sc*, pSCL4 cluster 12)		100 (SCLAV_p1129 – 1136)	NP	NP	NP	NA
	Bacteriocin-like BGC (*Sp*, cluster 4)		NP	NP	NP	100	NA
	Bacteriocin-like BGC (*Sp*, cluster 8)		NP	NP	NP	100	NA
	Bacteriocin-like BGC (*Sp*, cluster 12)		NP	NP	NP	100	NA
	Bacteriocin-like BGC (*Sp*, cluster 22)		NP	NP	NP	100	NA
**RiPP (Lantipeptide)**	AmfS	Morphogen	80 (SCLAV_4943 – 4973	80	80	NP	BGC0000496
	BD-12	Antibiotic	7 (SMC12)	14	NP	NP	BGC0001379
	Thioviridamide	Immunomodulator	NP	10	NP	NP	BGC0000625
	Venezuelin	Unknown	75 (SMCp7)	NP	NP	NP	BGC0000563

RiPP (Lassopeptide)	Lantipeptide-like BGC (*Sk*, cluster19)	Unknown	NP	NP	100	NP	NA
	Lantipeptide-like BGC (*Sc*, cluster 8)	Unknown	100 (SMC8)	NP	NP	NP	NA
	Lantipeptide-like BGC (*Sp*, cluster16)		NP	NP	NP	100	NA
	SSV-2083	Unknown	NP	NP	50	NP	BGC0000579
	Streptomycin	Antibiotic	NP	2	NP	NP	BGC0000717
	Lassopeptide-like BGC (*Sk*, cluster 12)	Unknown	NP	NP	100	NP	NA
	Lassopeptide-like BGC (*Sc*, pSCL4 cluster 3)		100 (SCLAV_p0400 – 0421)	NP	NP	NP	NA
**RiPP (Linaridin)**	Legonaridin	Unknown	NP	33	33	NP	BGC0001188
**Saccharides**	Istamycin	Antibiotic	NP	NP	NP	11	BGC000700
	Kanamycin	Antibiotic	NP	8	NP	NP	BGC0000703
	Paromomycin	Antibiotic	5 (SMC16)	NP	NP	NP	BGC0000712
	Streptomycin	Antibiotic	NP	2	13	NP	BGC0000717
**Siderophore**	**Desferrioxamine B**	Siderophore	NP	NP	NP	83	BGC0000940
			**100 (SMC6)**	**100**	**80**	NP	BGC0000941
	Siderophore-like BGCs (*Sj*, cluster 3; *Sk*, cluster 15)	Unknown	NP	100	97	NP	NA
	Siderophore-like BGCs (*Sj*, cluster 26; *Sc*, cluster 15)		78 (SCLAV_4677 – 4683)	100	NP	NP	NA
	Siderophore-like BGCs (*Sj*, cluster 48)		NP	100	NP	NP	NA
	Siderophore-like BGCs (*Sk*, cluster 27; *Sp*, cluster 10)		NP	NP	100	72	NA
	Siderophore-like BGCs (*Sc*, cluster 26)		100 (SMC23)	NP	NP	NP	NA
**Terpene**	Borrelidin	Angiogenesis inhibitor/antimicrobial	NP	4	4	NP	BGC0000031
	(-)-delta-cadinene	Unknown	100 (SMCp2 and SMCp3)	NP	NP	NP	BGC0000674
	Hopene	Membrane stability	69 (SMC17)	47	61	69	BGC0000663
	Isorenieratene	Carotenoid pigment	NP	NP	NP	100	BGC0000664

**Terpene**	**Naringenin**	Antimicrobial/antioxidant/antitumor	**100 (SCLAV_5491 – 5492)**	**100**	100	NP	BGC0001310
	Pactamycin	Antimicrobial/antimalarial	NP	NP	9	NP	BGC0000119
	Pentalenolactone	Antibiotic	NP	35	NP	NP	BGC0000653
	(+)-T-muurolol	Antifungal	80 (SMCp1)	NP	NP	NP	BGC0000675
	Terpene-like BGC (*Sj*, cluster 19; *Sk*, cluster 28)	Unknown	NP	100	97	NP	NA
	Terpene-like BGC (*Sj*, Cluster 45)	Unknown	NP	100	NP	NP	NA
	Terpene-like BGC (*Sc*, cluster 2)		100 (SMC4)	NP	NP	NP	NA
	Terpene-like BGC (*Sc*, pSCL4 cluster 4)		100 (SMCp4)	NP	NP	NP	NA
	Terpene-like BGC (*Sc*, pSCL4 cluster 7)		100 (SMCp9)	NP	NP	NP	NA
	Terpene-like BGC (*Sp*, cluster 11)		NP	NP	NP	100	NA
	Terpene-like BGC (*Sp*, cluster 17)		NP	NP	NP	100	NA
**T1PKS**	Enediyne (Neocarzinostatin)	Antitumor/antibiotic	15 (SMCp16)	6	NP	NP	BGC0000112
	Enediyne (Kedarcidin)	Antitumor	NP	NP	6	NP	BGC0000081
	Herboxidiene	Antitumor	8 (SMC21)	3	7	NP	BGC0001065
	JBIR-100	Antibiotic	80 (SMC1)	NP	NP	NP	BGC0001348
	Lobosamide (macrolactam)	Anti-trypanosomal	4 (SMCp13)	6	10	NP	BGC0001303
	Svaricin	Antifungal	NP	NP	6	NP	BGC0001382
**T2PKS**[l]	Lactonamycin	Antibiotic	3 (SCLAV_2306 – 2316)	NP	NP	3	BGC0000238
	Spore pigment	Protective pigment	75 (SMC15)	83	83	83	BGC0000271
	Rabelomycin	Antimicrobial (cytotoxic)	NP	NP	6	NP	BGC0000262

(Foot notes on following page)

[a] BGCs in bold font correspond with specific SMs detected in the current study (shown in Supplementary Table 5)

[b] Percent similarity of the specific BGC from each species to that of the corresponding BGC in column 2 is shown

[c] The parenthesis refer to the identifiers assigned to *S. clavuligerus* BGCs by Medema et al. (2010) or locus tags from StrepDB if BGCs were not identified in that study (http://strepdb.streptomyces.org.uk). SMCp: specialized metabolite cluster in plasmid pSCL4, SMC: specialized metabolite cluster in chromosome

[d] MIBiG BGC-ID: Minimal information about biosynthetic gene cluster-identification number

[e] NP: Not present

[f] NA: Not applicable

[g] pSCL4: The giant linear plasmid in *S. clavuligerus*

[h] NRPS: Non-ribosomal peptide synthetase

[i] PKS: Polyketide synthase

[j] T1PKS: Type 1 polyketide synthase

[k] RiPP: Ribosomally synthesized and post-translationally modified peptide

[l] T2PKS: Type 2 polyketide synthase

Supplementary Table S5. Specialized metabolites (SMs) detected with high confident and/or with associated biosynthetic gene clusters in *S. clavuligerus* (*Sc*), *S. jumonjinensis* (*Sj*) and *S. katsurahamanus* (*Sk*) using MS based metabolomics and GNPS analysis.

Name[a]	Species in which detected	Observed *m/z* [Adduct]	Molecular formula (weight, g/mol)	Cosine score	Shared peaks	Reference (*Sc*)[b]
(-)-Carveol	*Sc, Sj, Sk*	135.117 $[M - H_2O+H]^+$	$C_{10}H_{16}O$ (152.237)	0.96	13	This study only
**Desferrioxamine B**	*Sc, Sk*	561.361 $[M+H]^+$	$C_{25}H_{48}N_6O_8$ (560.693)	0.96	49	This study only
**Holomycin**	*Sc*	214.994 $[M+H]^+$	$C_7H_6N_2O_2S_2$ (214.257)	0.96	18	(Kenig and Reading, 1979)
**Naringenin**	*Sc, Sj*	271.062 $[M-H]^-$	$C_{15}H_{12}O_5$ (272.256)	0.95	19	(Álvarez-Álvarez et al., 2015)
Cuminyl alcohol	*Sc, Sj, Sk*	133.101 $[M - H_2O+H]^+$	$C_{10}H_{14}O$ (150.221)	0.95	16	This study only
**Tunicamycin C**	*Sc*	817.409 $[M+H]^+$	$C_{37}H_{60}N_4O_{16}$ (816.899)	0.93	75	(Kenig and Reading, 1979)
**Tunicamycin I-CH_2**	*Sc*	789.377 $[M+H]^+$	$C_{35}H_{56}N_4O_{16}$ (788.836)	0.93	67	(Martínez-Burgo et al., 2019)
**Clavulanic acid**	*Sc, Sk*	198.039 $[M-H]^-$	$C_8H_9NO_5$ (199.162)	0.92	29	(Reading and Cole, 1977)
**Tunicamycin I-2xCH_2**	*Sc*	775.361 $[M+H]^+$	$C_{34}H_{54}N_4O_{16}$ (774.809)	0.92	60	(Martínez-Burgo et al., 2019)
Hydroxyvalerenic acid	*Sc*	499.307 $[2M-H]^-$	$C_{15}H_{22}O_3$ (250.338)	0.91	5	This study only
Arthrobactin	*Sk*	477.256 $[M+H]^+$	$C_{20}H_{36}N_4O_9$ (476.527)	0.91	46	This study only
**Thiolutin**	*Sc*	229.010 $[M+H]^+$	$C_8H_8N_2O_2S_2$ (228.284)	0.91	41	This study only
**Tunicamycin B**	*Sc*	845.440 $[M+H]^+$	$C_{39}H_{64}N_4O_{16}$ (844.953)	0.91	63	(Kenig and Reading, 1979)
**Tunicamycin I**	*Sc*	803.393 $[M+H]^+$	$C_{36}H_{58}N_4O_{16}$ (802.872)	0.91	83	(Martínez-Burgo et al., 2019)
**Desferrioxamine E**	*Sc, Sj*	601.356 $[M+H]^+$	$C_{27}H_{48}N_6O_9$ (600.714)	0.89	37	(Álvarez-Álvarez et al., 2017)
(-)-Indolactam V	*Sc*	274.191 $[M - CO+H]^+$	$C_{17}H_{23}N_3O_2$ (301.39)	0.89	45	This study only
**Ectoine**	*Sc, Sj, Sk*	143.082 $[M+H]^+$	$C_6H_{10}N_2O_2$ (142.158)	0.89	7	This study only

Pentostatin	*Sc*	135.066 $[M+2H]^{2+}$	$C_{11}H_{16}N_4O_4$ (268.273)	0.89	19	This study only
**Tunicamycin A**	*Sc*	831.424 $[M+H]^{+}$	$C_{38}H_{62}N_4O_{16}$ (830.926)	0.87	77	(Kenig and Reading, 1979)
Cephamycin C	*Sc*	445.104 $[M-H]^{-}$	$C_{16}H_{22}N_4O_9S$ (446.431)	0.86	39	(Nagarajan et al., 1971)

[a] Corresponding BGCs for SMs indicated in bold font were also predicted in the current study using antiSMASH (shown in Supplementary Table 4)

[b] Relevant references are only included for metabolites previously detected in *S. clavuligerus*, as *S. jumonjinensis* and *S. katsurahamanus* have not been subjected to such metabolomics analysis until the current study

References:

Álvarez-Álvarez, R., Botas, A., Albillos, S.M., Rumbero, A., Martín, J.F., and Liras, P. (2015). Molecular genetics of naringenin biosynthesis, a typical plant secondary metabolite produced by *Streptomyces clavuligerus*. *Microb Cell Fact* 14, 178. doi: 10.1186/s12934-015-0373-7.

Álvarez-Álvarez, R., Martínez-Burgo, Y., Rodríguez-García, A., and Liras, P. (2017). Discovering the potential of *S. clavuligerus* for bioactive compound production: cross-talk between the chromosome and the pSCL4 mega-plasmid. *BMC Genomics* 18(1), 907. doi: 10.1186/s12864-017-4289-y.

Kenig, M., and Reading, C. (1979). Holomycin and an antibiotic (MM 19290) related to tunicamycin, metabolites of *Streptomyces clavuligerus*. *J Antibiot* (Tokyo) 32(6), 549-554.

Martínez-Burgo, Y., Santos-Aberturas, J., Rodríguez-García, A., Barreales, E.G., Tormo, J.R., Truman, A.W., et al. (2019). Activation of Secondary Metabolite Gene Clusters in *Streptomyces clavuligerus* by the PimM Regulator of *Streptomyces natalensis*. *Front Microbiol* 10, 580. doi: 10.3389/fmicb.2019.00580.

Nagarajan, R., Boeck, L.D., Gorman, M., Hamill, R.L., Higgens, C.E., Hoehn, M.M., et al. (1971). Beta-lactam antibiotics from *Streptomyces*. *J Am Chem Soc* 93(9), 2308-2310. doi: 10.1021/ja00738a035.

Reading, C., and Cole, M. (1977). Clavulanic acid: a beta-lactamase-inhibiting beta-lactam from *Streptomyces clavuligerus*. *Antimicrob Agents Chemother* 11(5), 852-857. doi: 10.1128/aac.11.5.852.

Supplementary Table S6. Other potential specialized metabolites detected in *S. clavuligerus* (*Sc*), *S. jumonjinensis* (*Sj*) and *S. katsurahamanus* (*Sk*) using MS based metabolomics and GNPS analysis.

Name	Species in which detected	Observed *m/z* [Adduct]	Molecular formula (Weight, g/mol)	Cosine Score	Shared peaks
Allantoin	*Sc, Sk*	157.036 $[M-H]^-$	$C_4H_6N_4O_3$ (158.117)	0.96	5
Tunicamycin derivatives	*Sc*	568.287 $[M-C_8H_{15}NO_6+H]^+$<br>582.303 $[M-C_8H_{15}NO_6+H]^+$<br>610.333 $[M-C_8H_{15}NO_6+H]^+$	$C_{35}H_{56}N_4O_{16}$ (788.836)<br>$C_{36}H_{58}N_4O_{16}$ (802.872)<br>$C_{38}H_{62}N_4O_{16}$ (830.926)	0.90<br>0.89<br>0.82	66<br>67<br>75
(-)-Caryophyllene oxide	*Sc, Sj, Sk*	221.19 $[M+H]^+$	$C_{15}H_{24}O$ (220.356)	0.89	31
L-Saccharopine	*Sc, Sj*	277.155 $[M+H]^+$	$C_{11}H_{20}N_2O_6$ (276.2863)	0.87	11
Dehydroxynocardamine	*Sc, Sj*	585.361 $[M+H]^+$	$C_{27}H_{48}N_6O_8$ (584.715)	0.87	58
Isoalantolactone	*Sc*	215.143 $[M -H_2O+H]^+$	$C_{15}H_{20}O_2$ (232.323)	0.87	38
Valerenic acid	*Sc, Sj, Sk*	217.159 $[M-H_2O+H]^+$	$C_{15}H_{22}O_2$ (234.339)	0.87	36
Costunolide	*Sc, Sj, Sk*	233.154 $[M+H]^+$	$C_{15}H_{20}O_2$ (232.323)	0.84	34
Genipin	*Sk*	209.092 $[M -H_2O+H]^+$	$C_{11}H_{14}O_5$ (226.226)	0.83	11
Maesopsin	*Sc*	287.057 $[M-H]^-$	$C_{15}H_{12}O_6$ (288.255)	0.82	27
Endothal	*Sc, Sk*	141.055 $[M -CH_2O_2+H]^+$	$C_8H_{10}O_5$ (186.163)	0.81	4
Indolactam derivative related to lyngbyatoxin A	*Sc*	424.296 $[M+H]^+$<br>396.301 $[M-CO+H]^+$	$C_{26}H_{37}N_3O_2$ (423.591)	0.81<br>0.89	59<br>63
Lyngbyatoxin A (putative)	*Sc, Sj, Sk*	438.311 $[M+H]^+$	$C_{27}H_{39}N_3O_2$ (437.628)	0.78	60
Parthenolide	*Sc, Sj, Sk*	249.148 $[M+H]^+$	$C_{15}H_{20}O_3$ (248.317)	0.76	34
Brefeldin A	*Sj, Sk*	245.154 $[M -2H_2O+H]^+$	$C_{16}H_{24}O_4$ (280.36)	0.76	35
Stylopine	*Sk*	149.06 $[M -C_{10}H_9O_2N+H]^+$	$C_{19}H_{17}NO_4$ (323.348)	0.74	3
Cordycepin	*Sk*	269.124 $[M+NH_4]^+$	$C_{10}H_{13}N_5O_3$ (251.246)	0.73	19
Fumagillin	*Sk*	233.117 [233.1]	$C_{26}H_{34}O_7$ (458.551)	0.72	22
Aurapten	*Sc*	175.05 $[M -C_9H_{16}+H]^+$	$C_{19}H_{22}O_3$ (298.382)	0.71	8
Imazapic	*Sc, Sj, Sk*	258.124 $[M -H_2O+H]^+$	$C_{14}H_{17}N_3O_3$ (275.308)	0.71	7

Sophocarpine	*Sc*	150.136 $[M -C_5H_7ON+H]^+$	$C_{15}H_{22}N_2O$ (246.354)	0.69	19
Artemisinin	*Sc, Sj, Sk*	283.152 $[M+H]^+$	$C_{15}H_{22}O_5$ (282.336)	0.68	30
Oseltamivir acid	*Sc*	197.078 $[M -C_5H_{12}O+H]^+$	$C_{14}H_{24}N_2O_4$ (284.356)	0.67	6
Tomatidine	*Sj*	416.353 $[M+H]^+$	$C_{27}H_{45}NO_2$ (415.662)	0.66	26
Strobilactone A	*Sc, Sj, Sk*	265.148 $[M-H]^-$	$C_{15}H_{22}O_4$ (266.337)	0.66	9
Indole	*Sk*	118.065 $[M+H]^+$	C_8H_7N (117.151)	0.65	3
Bisucaberin	*Sc*	401.24 $[M+H]^+$	$C_{18}H_{32}N_4O_6$ (400.476)	0.65	41
Neoandrographolide	*Sc*	479.266 $[M-H]^-$	$C_{26}H_{40}O_8$ (480.598)	0.64	12
Glabridin	*Sk*	189.095 $[M -C_8H_8O_2+H]^+$	$C_{20}H_{20}O_4$ (324.380)	0.62	22
Anemonin	*Sk*	193.061 $[M+H]^+$	$C_{10}H_8O_4$ (192.171)	0.61	13

Supplementary Figure S1. Mass spectrometric (MS) detection of clavulanic acid (CA) in producing *Streptomyces* species. MS analysis of 96-hour SA culture supernatants (as shown in Fig. 2B) from *S. jumonjinensis, S. katsurahamanus* and *S. clavuligerus* showing the spectra of peaks corresponding imidazole derivatized CA $[M+H]^+$ (m/z = 224) and the fragmented product $[M\text{-imidazole}]^+$ (m/z = 156), which are indicated by (*).

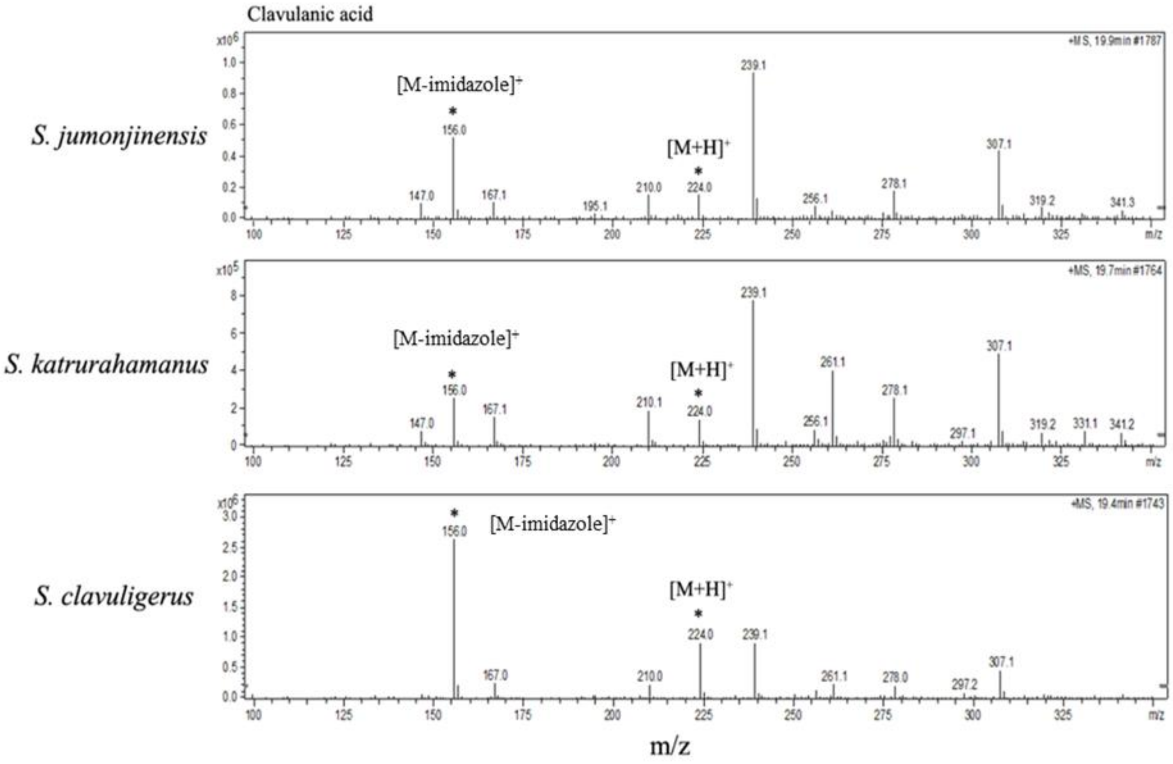

Supplementary Figure S2. Phylogeny of clavulanic acid (CA) producing (red stars) and non-producing (black stars) *Streptomyces* species known to contain CA or CA-like biosynthetic gene clusters (BGCs), respectively. Certain *Streptomyces* species known to produce cephamycin C (from Figure 3) were also included (blue stars) along with some others for comparison. The maximum likelihood tree was built using 16S rRNA gene sequences. Bootstrap values of >75% were obtained at the respective branch points comprising the marked species based on 100 repetitions. The accession numbers for the genome sequences or the 16s rRNA genes used in the analysis are included after the names each species. *Saccharomonospora viridis* DSM 43017 was included as an outcrop as it does not produce CA but contains a CA-like BGC. The scale bar indicates the number of nucleotide substitutions per site.

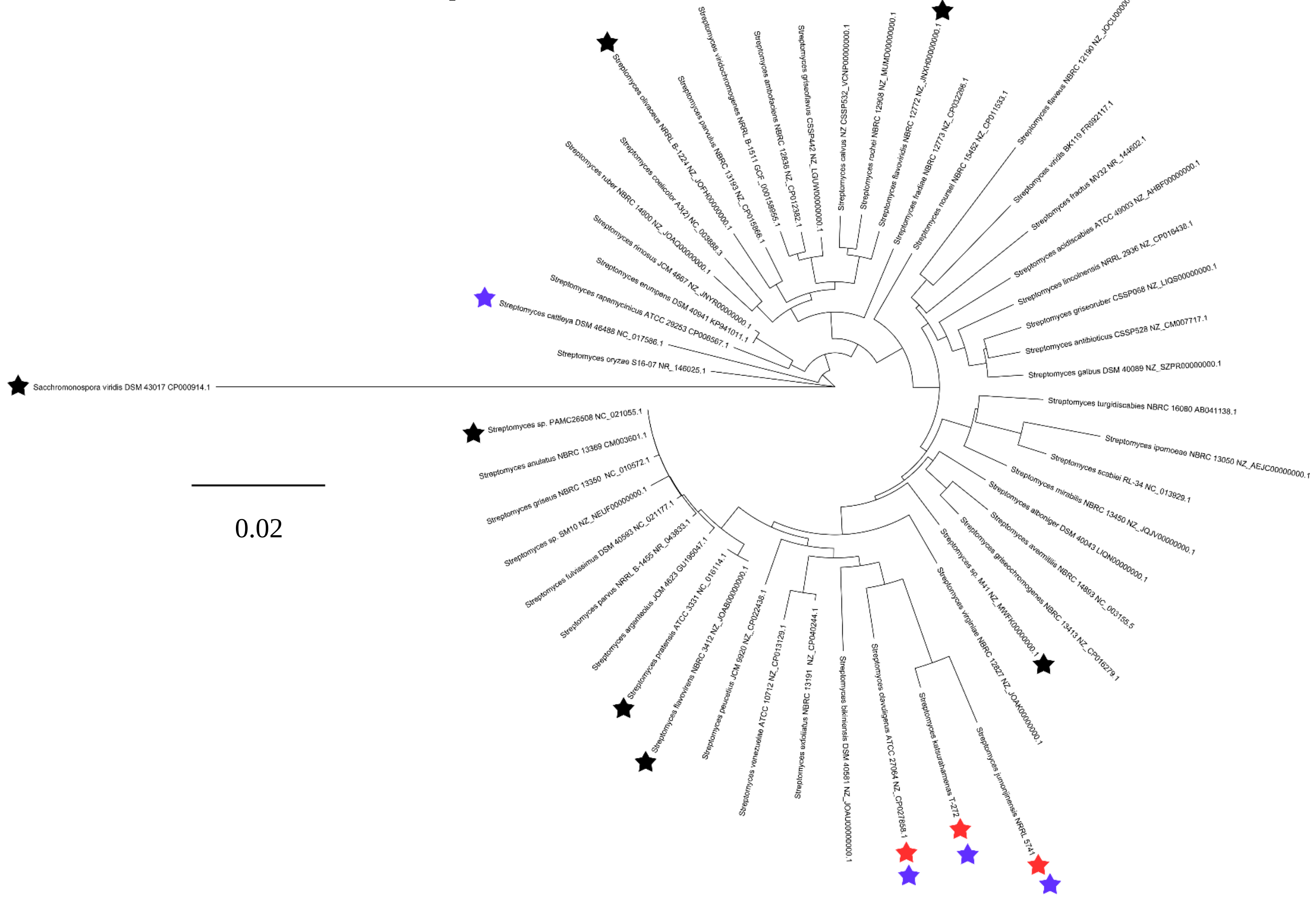

Supplementary Figure S3. Mass spectrometric (MS) detection of clavulanic acid (CA) and 2-Hydroxymethylclavam (2-HMC) in the wt, *ΔnocE* and *ermE*p*-*nocE* strains of *S. clavuligerus*. MS analysis of 96-hour soy culture supernatants (from Fig. 4B) showing the spectra of peaks corresponding to CA and 2-HMC. The major peaks due to imidazole derivatized CA $[M+H]^+$ (m/z = 224) and the fragmented product $[M\text{-imidazole}]^+$ (m/z = 156), and imidazole derivatized 2HMC $[M+H]^+$ (m/z = 212) and the fragmented product $[M\text{-imidazole}]^+$ (m/z = 144) are indicated by (*).

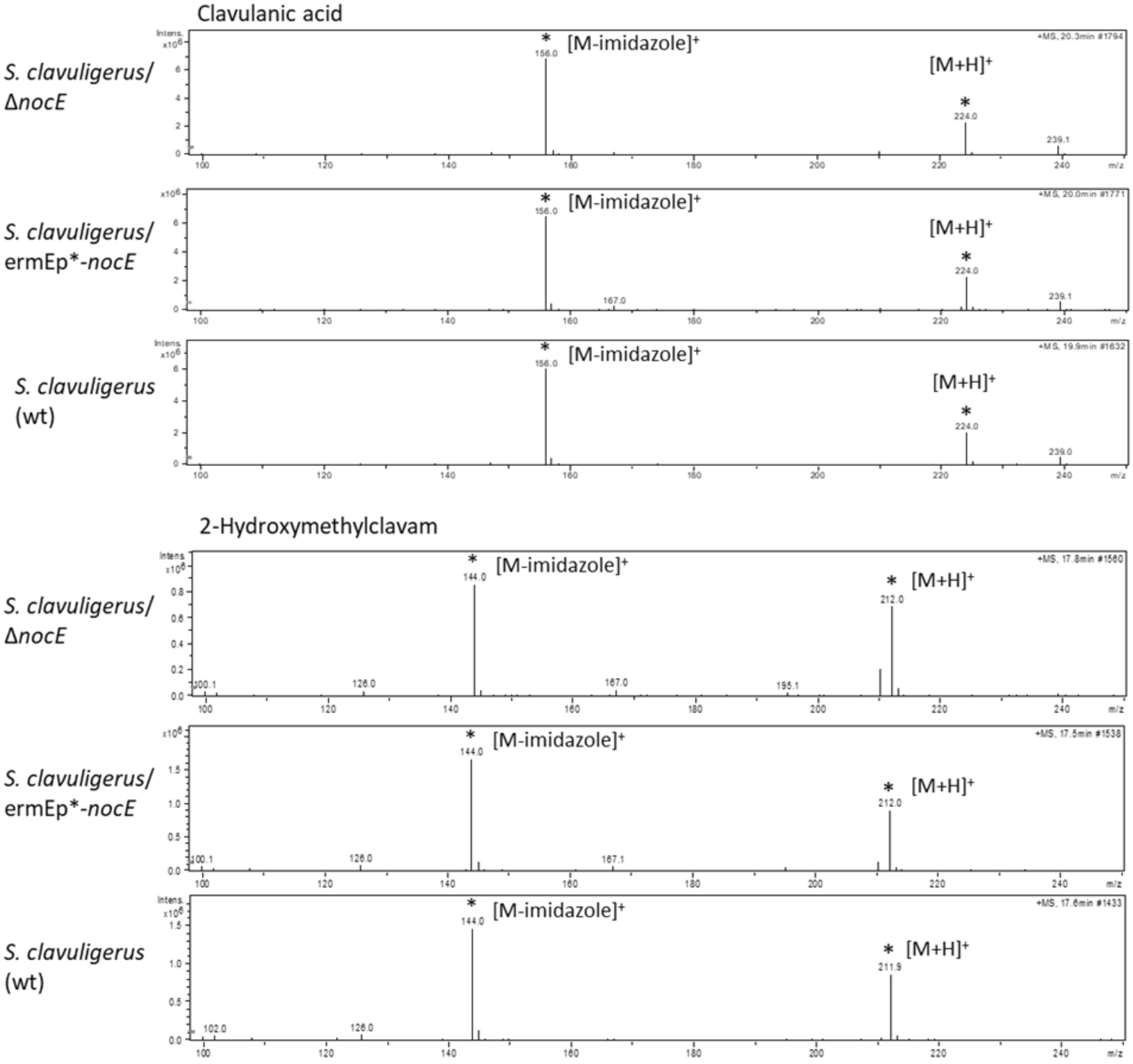

Supplement: Supplementary file 1 [file Data_Sheet_1.pdf]
